# Self‐Amplified Activatable Fluorescent Probe for Early Diagnosis of Acute Kidney Injury and High‐Throughput Screening of Natural Protectants

**DOI:** 10.1002/advs.77162

**Published:** 2026-08-13

**Authors:** Yahui Chen, Meihong Liu, Mengchao Ding, Ye Liu, Chuanxu Zhu, Mingye Gong, Zhixuan Huang, Yi Zhang, Wei Xue, Youyong Yuan, Kewei Wang

**Affiliations:** ^1^ Key Laboratory of Biomaterials of Guangdong Higher Education Institutes Department of Biomedical Engineering and MOE Key Laboratory of Tumor Molecular Biology Jinan University Guangzhou People's Republic of China; ^2^ Guangdong Provincial Key Laboratory of Spine and Spinal Cord Reconstruction The Fifth Affiliated Hospital of Jinan University (Heyuan Shenhe People's Hospital) Jinan University Heyuan People's Republic of China; ^3^ School of Biomedical Sciences and Engineering Guangzhou International Campus South China University of Technology Guangzhou People's Republic of China

**Keywords:** acute kidney injury, chrysin, high‐throughput screening, self‐amplifying fluorescent sensor, self‐immolative

## Abstract

Early diagnosis of acute kidney injury (AKI) and identification of effective therapeutics remain significant clinical challenges. Herein, we developed an activatable near‐infrared fluorescent nanoprobe (denoted PS‐QRC) by encapsulating a self‐amplifying fluorescent sensor (QRCy) within kidney‐targetable *L*‐serine‐functionalized ultrasmall poly(amidoamine) (PAMAM‐Ser) dendrimers. Upon activation by reactive oxygen species (ROS), QRCy undergoes self‐fragmentation, releasing four fluorophores to amplify the fluorescence signal, affording an ultra‐low detection limit of 81 nM for hydrogen peroxide. The *L*‐serine modification enables specific binding of PS‐QRC to kidney injury molecule‐1 (KIM‐1), which is overexpressed in injured renal tubules, thereby promoting renal accumulation and reducing background interference. Impressively, PS‐QRC allows noninvasive real‐time imaging of cisplatin‐induced AKI in mice as early as 12 h, significantly earlier than conventional serum biochemical and histopathological analyses. Furthermore, a high‐throughput screening platform was established using this nanoprobe to evaluate natural products, from which chrysin was identified as a promising renoprotective agent. Chrysin demonstrated both in vitro and in vivo renoprotective effects, with efficacy comparable to the established antioxidant *N*‐acetylcysteine. Moreover, PS‐QRC enabled quantitative monitoring of chrysin's dose‐dependent therapeutic response. Overall, this integrated probe provides a sensitive and reliable tool for early AKI diagnosis and the high‐throughput discovery of novel renoprotective drugs.

## Graphical abstract

The PS‐QRC nanoprobe employs L‐serine as a targeting ligand for kidney injury molecule‐1 (KIM‐1) to achieve accumulation in injured renal tissues. Upon activation by reactive oxygen species (ROS), it undergoes a self‐amplifying cascade to release fluorophores for near‐infrared fluorescence (NIRF) imaging, enabling ultra‐early diagnosis of acute kidney injury. Furthermore, this platform facilitates high‐throughput screening, identifying chrysin as a renoprotective agent.

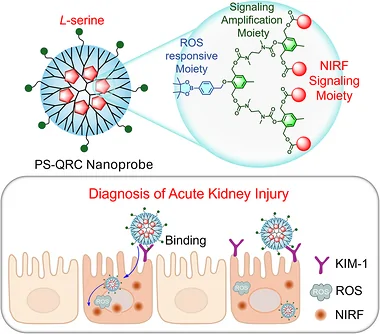

## 1 Introduction

Acute kidney injury (AKI), a disease characterized by rapid renal function decline and often linked to nephrotoxic agent exposure, has emerged as a critical global clinical concern owing to its potential to induce multiple organ dysfunction and its substantial worldwide disease burden [1, 2]. Early and accurate diagnosis followed by timely protective interventions is an effective approach to preventing AKI. Currently, the clinical diagnosis of AKI primarily relies on serum biochemical markers, with serum creatinine (sCr) and blood urea nitrogen (BUN) as the most commonly used indicators in clinical practice [3, 4]. However, these markers often lack sensitivity and specificity for early renal dysfunction and typically remain within the normal range until up to 50% of the glomerular filtration rate is lost [5, 6]. This delay in abnormal expression means that these markers cannot reflect the early subtle changes in renal function, often leading to missed opportunities for early intervention. In addition to biochemical markers, traditional imaging techniques are also used as auxiliary diagnostic tools for AKI, including computed tomography, magnetic resonance imaging, and ultrasound [7, 8, 9]. However, these imaging methods are still insufficient for the early diagnosis of AKI at the molecular and cellular levels. Early AKI is primarily characterized by subtle pathological changes at the cellular level, such as renal tubular epithelial cell injury, apoptosis, or microcirculatory disturbance, which cannot be effectively identified by traditional imaging techniques that focus on macrostructural changes [10, 11]. Therefore, the inadequacies of current diagnostic methods highlight the urgent need for the development of highly sensitive and specific early diagnostic technologies for AKI.

Molecular optical imaging enables noninvasive, real‐time visualization of disease onset and progression in living organisms with high spatiotemporal resolution [12, 13, 14]. It has emerged as a valuable tool for in situ monitoring of molecular events associated with kidney injury [15, 16, 17]. A variety of fluorescent probes have been developed for detecting AKI‐related biomarkers at the molecular level, including kidney injury molecule‐1 (KIM‐1) [18], *N*‐acetyl‐*β*‐Dglucosaminidase (NAG) [19], reactive oxygen species (ROS) [20, 21, 22], cell apoptosis executor caspase‐3 (Casp‐3) [5], and hypochlorous acid (HOCl) [23]. However, most currently developed probes rely on one‑to‑one activation between a biomarker and a fluorophore, resulting in intrinsically low signal intensity that is easily overwhelmed by background noise, especially given the extremely low concentrations and subtle changes of associated biomarkers in the early stage of AKI [18]. Moreover, poor renal targeting and rapid clearance further limit their ability to monitor early AKI progression [24, 25]. In contrast, self‐immolative chemistry provides an effective solution to the signal sensitivity issue of traditional probes [26, 27]. Upon interaction with a single target analyte, self‐immolative probes undergo a domino‐like fragmentation reaction, generating multiple fluorescent species from one probe molecule [28, 29, 30]. This intrinsic signal amplification mechanism significantly enhances detection sensitivity, enabling the accurate identification of low‐abundance biomarkers in the early stage of AKI. Therefore, the development of fluorescent probes that integrate renal‐targeting capabilities with built‐in self‐immolative signal amplification functions represent a critical research direction for early and accurate diagnosis of AKI.

On the other hand, enhancing patient outcomes necessitates the early detection of AKI be coupled with efficacious therapeutic interventions. Unfortunately, no AKI‑specific therapies are currently available, and clinicians must rely on supportive measures such as fluid management, electrolyte balance, and renal replacement therapy [31, 32]. Therefore, effective and targeted therapeutic strategies for AKI are urgently needed. Given the complex pathophysiological mechanisms of AKI, including oxidative stress, inflammation, and tubular cell injury, natural products have emerged as attractive sources of potential renoprotective agents owing to their structural diversity and multi‐targeting properties [33, 34, 35, 36]. However, the complexity of natural product libraries often renders traditional screening approaches labor‐intensive and time‐consuming. Thus, fluorescence‐based high‐throughput screening (HTS) has become a powerful strategy for accelerating the discovery of bioactive natural product components [37, 38, 39, 40]. Although several fluorescent probe‐based HTS methods have been developed, their utility in AKI drug discovery remains limited by the lack of probes with high sensitivity, excellent selectivity, and a robust signal‐to‐noise ratio. Therefore, integrating advanced fluorescent probes with HTS technologies holds significant promise for identifying novel natural product‐derived therapeutics for AKI.

Herein, we designed and fabricated an activatable near‐infrared (NIR) fluorescent nanoprobe denoted as PS‐QRC by loading a hydrogen peroxide (H_2_O_2_)‐triggered self‐amplified fluorescent sensor QRCy into *L*‐serine‐modified ultrasmall poly(amidoamine) (PAMAM) dendrimers for noninvasive, real‐time imaging of renal cell injury in living mice during the early stage of cisplatin‐induced AKI. The core QRCy fluorescent sensor incorporates a rationally designed self‐immolative linker, which can be selectively activated by a single ROS stimulus, triggering a sequential self‐fragmentation process and the concomitant release of four fluorescent moieties (Scheme 1A). This unique structural design enables intrinsic fluorescence signal self‐amplification without external auxiliary conditions, effectively overcoming the limitation of weak signal output in conventional fluorescent probes and significantly enhancing detection sensitivity for early AKI‐related ROS fluctuations. Meanwhile, *L*‐serine functionalization of the ultrasmall PAMAM dendrimer carrier not only facilitates efficient renal metabolism of the nanoprobe, but also mediates its specific binding to KIM‐1 (Scheme 1B). Such precise kidney‐targeted delivery markedly promotes local enrichment of PS‐QRC in injured kidneys, while simultaneously minimizing nonspecific adsorption in other healthy organs and background fluorescence interference. Beyond noninvasive real‐time fluorescence imaging of early cisplatin‐induced AKI in living models, this tailor‐made nanoprobe also serves as a robust and reliable platform for high‐throughput screening of natural products with potential renoprotective activities. Collectively, the constructed PS‐QRC nanoprobe integrates exceptional renal targeting capacity and ROS‐driven signal self‐amplification properties, offering a promising and versatile tool for early, accurate AKI diagnosis and rapid discovery of novel renoprotective drug candidates.

**SCHEME 1:**
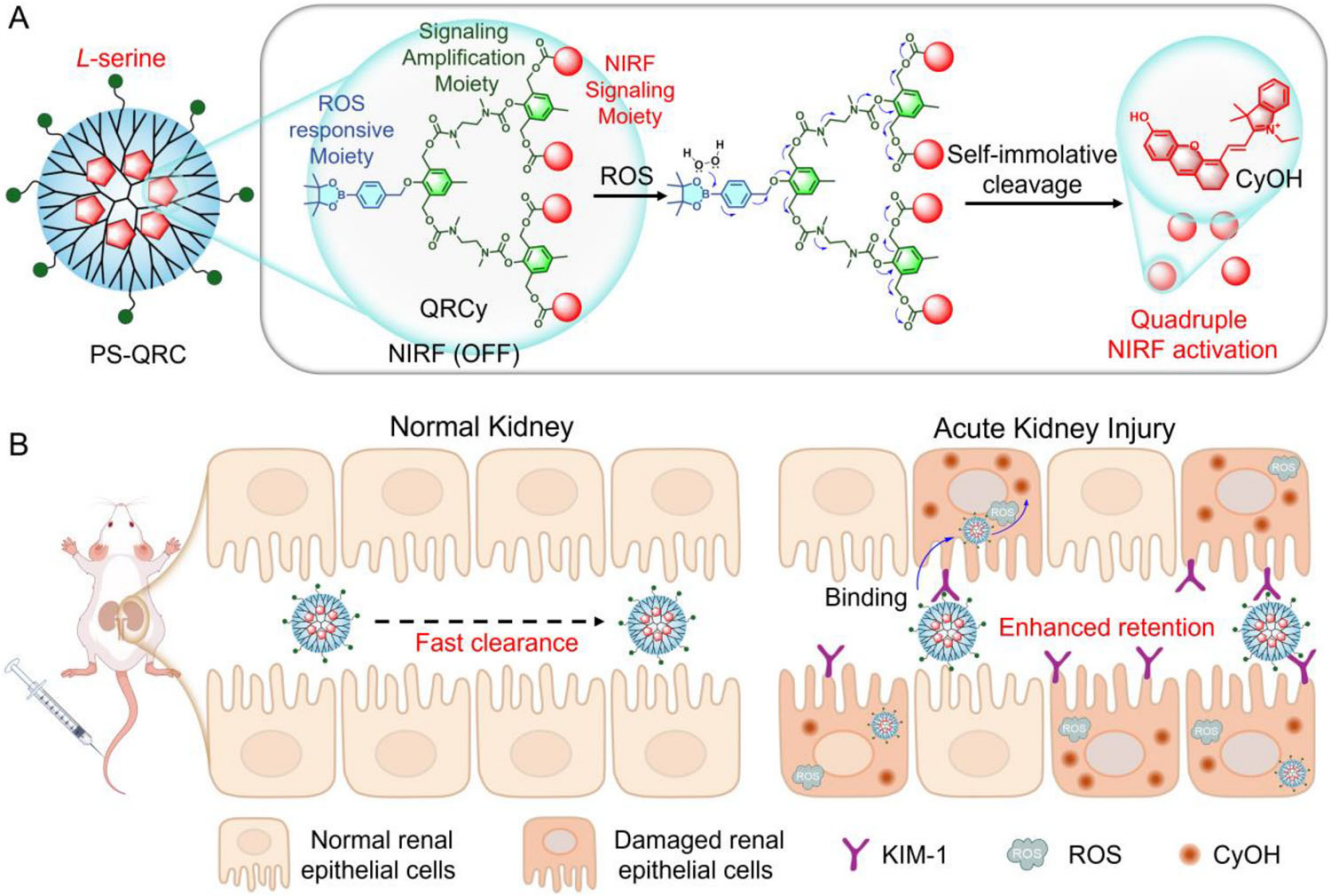
(A) Schematic illustration of the signal amplification mechanism of the PS‐QRC fluorescent nanoprobe. (B) Schematic diagram illustrating the synergistic mechanism of PS‐QRC for early AKI diagnosis via H_2_O_2_‐responsive activation and KIM‐1‐targeted renal accumulation.

## 2 Results and Discussion

### 2.1 Design and Synthesis of RCy, DRCy, and QRCy

To improve the detection efficacy of early biomarkers within the AKI microenvironment and enhance the performance of in vivo AKI fluorescence imaging, a series of rationally designed signal‐amplifying fluorescent probes has been developed. These probes incorporate the hemicyanine fluorophore (CyOH) as a NIR signal reporter due to its high fluorescence quantum yield and structural versatility [28, 41], and a phenylboronic acid group that functions as a selective H_2_O_2_‐responsive trigger (Figure 1A). The operational mechanism is founded on H_2_O_2_‐induced boronate ester deprotection, which initiates sequential cleavage of the probe architecture and the controlled liberation of the active CyOH fluorophore. This strategy enables the real‐time monitoring of pathological ROS fluctuations within injured renal tissue, establishing a robust platform for sensitive early‐stage AKI fluorescence imaging.

**FIGURE 1:**
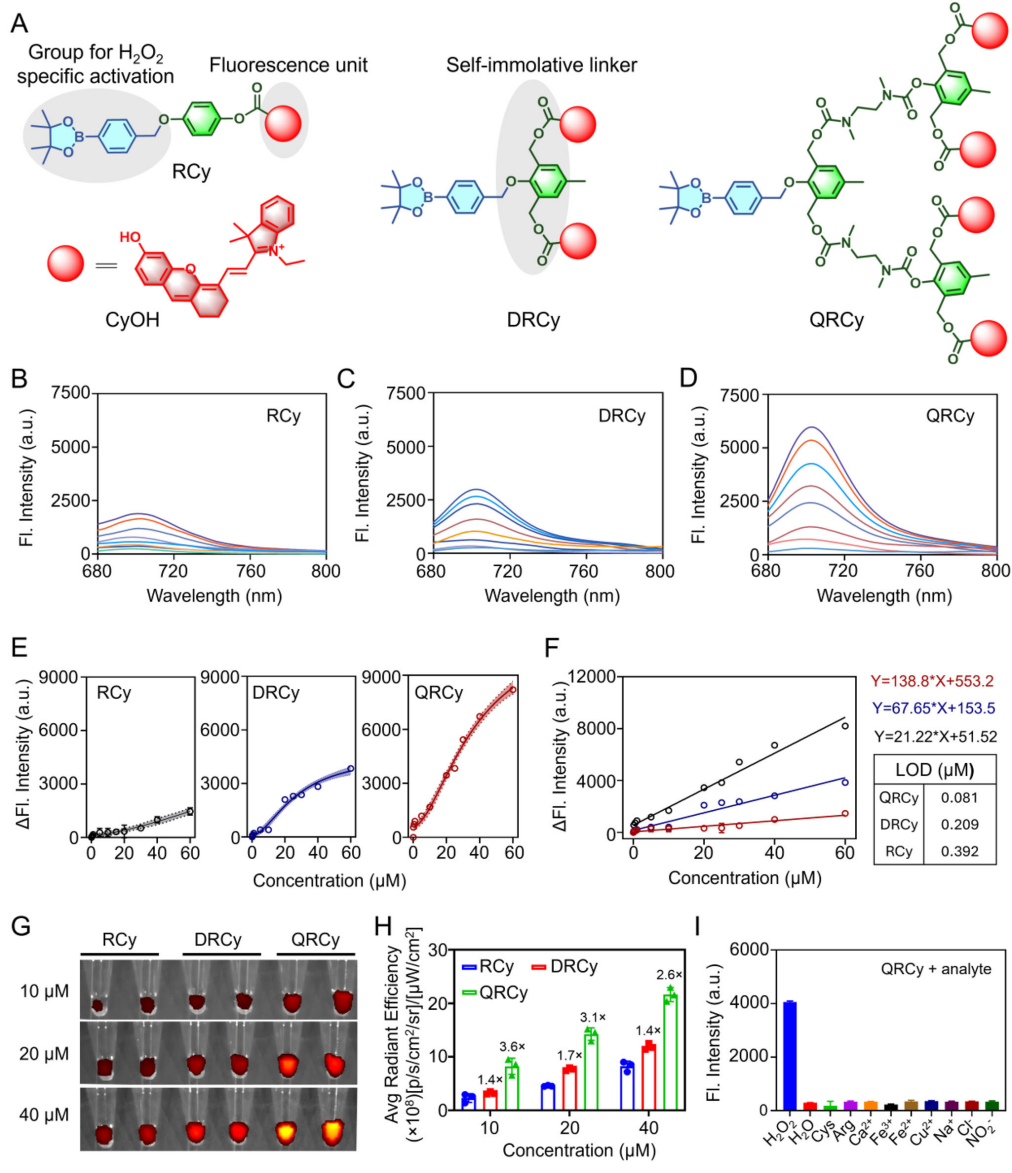
Synthesis and H_2_O_2_‐responsive performance of RCy, DRCy and QRCy. (A) Chemical structure diagrams of RCy, DRCy, and QRCy. Fluorescence spectra of RCy (B), DRCy (C), and QRCy (D) in the presence of H_2_O_2_ at different concentrations (0, 0.1, 0.5, 1, 5, 10, 20, 40, 80 µM). (E) Fluorescence intensity of RCy, DRCy, and QRCy incubated with different concentrations of H_2_O_2_ (*n* = 3). (F) The linear curves obtained by the fluorescence intensity of RCy, DRCy, and QRCy incubated with different concentrations of H_2_O_2_ (*n* = 3). Fluorescence images (G) and intensity analysis (H) of RCy, DRCy, and QRCy after treatment with different concentrations of H_2_O_2_ (*n* = 3). (I) Fluorescence responses of QRCy toward various analytes (*n* = 3). Data are presented as the mean ± SD; *n* = 3.

The synthetic route for CyOH is depicted in Scheme S1, and the purified product was fully characterized by ^1^H nuclear magnetic resonance (^1^H NMR), ^13^C NMR, and mass spectrometry (MS) to verify its chemical structure, with the corresponding spectra displayed in Figures S1–S6. To systematically validate the feasibility of the signal amplification strategy, three probes, including a reference probe (RCy) and two self‐amplifying probes (DRCy and QRCy), were designed and synthesized for comparative analysis. For the reference probe RCy, one molecule of H_2_O_2_ activates a single CyOH unit, which results in no accompanying signal amplification, whereas DRCy and QRCy are functionalized with self‐immolative linkers that undergo complete self‐fragmentation upon reaction with a single H_2_O_2_ molecule, which in turn liberates two and four CyOH fluorophores to deliver twofold and fourfold fluorescence signal enhancement that markedly improves imaging sensitivity for early‐stage AKI. All probes were prepared via stepwise synthetic routes, while the detailed synthetic pathways for each probe are detailed in Schemes S2–S4, and all target probes alongside their corresponding intermediates were comprehensively characterized by ^1^H NMR, ^13^C NMR, and MS, with the relevant spectral data presented in Figures S7–S21.

### 2.2 Spectral Response of Probes to H_2_O_2_ In Vitro

To comprehensively assess the optical properties of CyOH and the H_2_O_2_‐responsive performance of the designed probes (RCy, DRCy, and QRCy), we first conducted systematic characterizations using UV–vis absorption and fluorescence emission spectroscopy. CyOH exhibited a distinct absorption peak at 660 nm, with a dominant fluorescence emission maximum at 700 nm (Figures S22A,B). Owing to its NIR emission, this fluorophore effectively mitigates tissue autofluorescence and reduces photon scattering, which facilitates a high signal‐to‐background ratio (SBR) imaging in deep‐tissue regions. Subsequently, we investigated the dynamic changes in the fluorescence spectra of the three probes before and after incubation with H_2_O_2_. In the absence of H_2_O_2_, all probes displayed negligible fluorescence emission at 700 nm (Figure 1B–D), a result attributable to the effective suppression of intramolecular charge transfer by the 4‐(bromomethyl)phenylboronic acid pinacol ester‐based trigger moiety. In contrast, upon treatment with H_2_O_2_, all probes exhibited a substantial increase in fluorescence intensity at 700 nm, which is mechanistically explained by the H_2_O_2_‐mediated cleavage of the boronate ester linkage, triggering the release of the free CyOH fluorophore. Notably, the magnitude of fluorescence enhancement was observed to vary considerably among the three distinct probes. Specifically, following treatment with 80 µM H_2_O_2_, the fluorescence intensity of QRCy was enhanced by approximately 19.6‐fold, a value that was markedly higher than those recorded for DRCy (11.4‐fold) and RCy (7.0‐fold). These findings clearly demonstrate the superior signal‐amplification capabilities of the self‐immolative scaffold‐integrated probes (DRCy and QRCy) in comparison with the non‐amplified reference probe RCy.

The limit of detection (LOD) was systematically investigated in this study as a pivotal quantitative parameter for evaluating the intrinsic sensing sensitivity of responsive fluorescent probes, especially for the selective and accurate detection of low abundance endogenous H_2_O_2_, which is closely linked to the early pathogenesis of AKI. To assess and compare the sensing sensitivity of the three synthetic probes RCy, DRCy, and QRCy, we quantitatively analyzed the dynamic changes in their fluorescence emission intensities upon incubation with a series of H_2_O_2_ concentrations. The corresponding concentration‐dependent fluorescence response profiles are presented in Figure 1E, which illustrate the distinct responsiveness of each probe to increasing H_2_O_2_ concentrations. Linear regression fitting of the fluorescence intensity versus H_2_O_2_ concentration plots (Figure 1F) allowed the calculation of the LOD as 81 nm for QRCy, 209 nm for DRCy, and 392 nM for RCy, demonstrating a clear sensitivity gradient that correlates with the structural differences among the probe scaffolds. Remarkably, the LOD of 81 nM achieved by QRCy is substantially lower than those of the other two probes, which directly demonstrates its superior sensing sensitivity toward trace levels of H_2_O_2_ in the complex renal microenvironment associated with the early stage of AKI.

To further validate the concentration‐dependent response and signal amplification efficiency of these probes, fluorescence imaging and quantitative fluorescence intensity analysis were performed at H_2_O_2_ concentrations of 10, 20, and 40 µM. As shown in Figure 1G, the fluorescence signals of RCy, DRCy, and QRCy gradually increased with elevated H_2_O_2_ concentrations, and QRCy exhibited the most intense fluorescence at 40 µM H_2_O_2_. Notably, QRCy already exhibited a discernible fluorescence signal at 10 µM H_2_O_2_, whereas the signal from RCy remained very weak, providing a clear visual demonstration of the superior sensitivity of QRCy toward low H_2_O_2_ concentrations. Quantitative analysis (Figure 1H) confirmed a positive correlation between fluorescence intensity and H_2_O_2_ concentration, and QRCy displayed significantly stronger fluorescence enhancement than DRCy and RCy at all tested concentrations. Specifically, at 10 µM H_2_O_2_, the fluorescence intensity of QRCy was approximately 4.2‐fold that of RCy and 2.6‐fold that of DRCy. Furthermore, all probes showed negligible responses to potential interfering analytes (Figure 1I and Figure S23A–C), confirming their high selectivity for H_2_O_2_. Taken together, this signal self‐amplifying design, as exemplified by QRCy, endows the probe with prominent signal amplification capability, rendering it a promising tool for the sensitive and specific early detection of organ injuries, such as AKI, inflammatory bowel disease, and liver injury, all of which share similar pathological characteristics, particularly excessive ROS accumulation [42]

### 2.3 Kidney‐Targeted Delivery of Fluorescent Probes via PAMAM Nanocarriers

Fluorescent probes employed for AKI diagnosis often suffer from insufficient renal‐targeting efficiency or rapid clearance in vivo, which severely restricts the real‐time and continuous monitoring of renal function during AKI progression [17, 43]. Recent developments in nanoparticle‐based drug delivery systems have highlighted the unique physicochemical properties of nanoparticles that enable targeted delivery of therapeutic and diagnostic agents to the kidney [44, 45, 46]. In this study, PAMAM were selected as the nano‐carrier platform owing to their favorable physicochemical characteristics for renal targeting and delivery [47, 48]. Specifically, such dendrimers possess an ultrasmall hydrodynamic diameter below 10 nm, which is well within the glomerular filtration threshold for soft and deformable nanoparticles [49]. This characteristic allows them to efficiently traverse the glomerular filtration barrier and accumulate in renal tissue. Furthermore, their intrinsic positive surface charge facilitates glomerular filtration and enhances renal retention, thereby prolonging the in vivo half‐life of the loaded probes and improving overall imaging performance. To further improve the targeting specificity toward injured renal tissues, the surface of PAMAM was functionalized with *L*‐serine, which can specifically recognize KIM‐1, a biomarker that is significantly upregulated in the tubular epithelium during the early stages of AKI (Figure 2A) [50, 51, 52]. Dynamic light scattering (DLS) analysis confirmed that the resultant *L*‐serine‐modified PAMAM conjugates (PS) exhibited an average hydrodynamic diameter of 8.3 ± 1.9 nm, while transmission electron microscopy (TEM) imaging revealed that these PS conjugates displayed a uniformly dispersed, spherical morphology (Figure 2B). Meanwhile, zeta potential measurements recorded a surface charge value of +31.7 mV for PS (Figure 2C), which is consistent with the intrinsic high positive charge of PAMAM dendrimers and contributes to their excellent renal retention capability.

**FIGURE 2:**
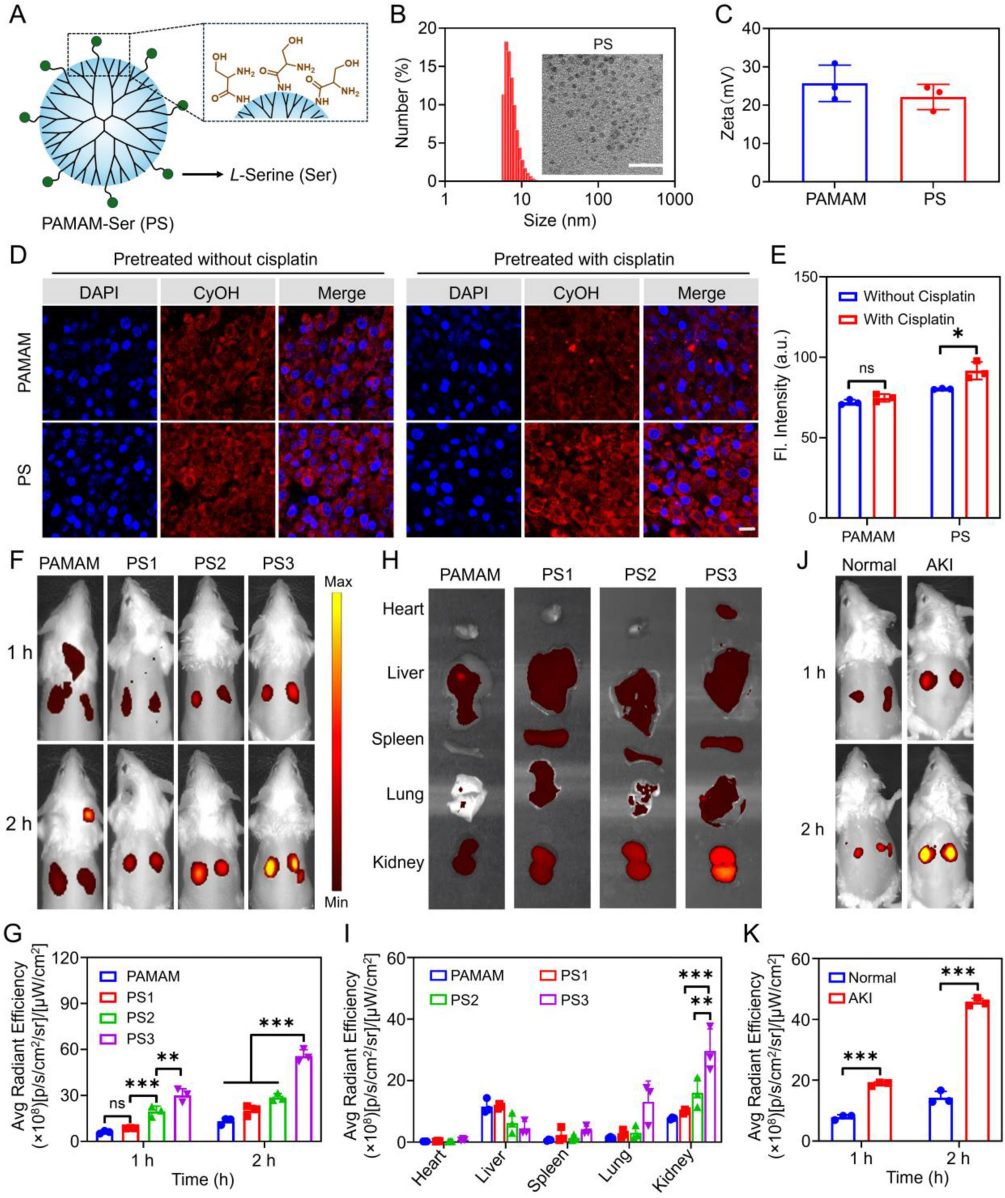
Construction and screening of PS nanocarriers with injured kidney‐targeting capability. (A) Chemical structure of PS. (B) Particle size distribution and TEM image of PS nanoparticles. (C) Zeta potentials of PAMAM and PS (*n* = 3). (D) Representative fluorescence microscopy images of HK‐2 cells incubated with PAMAM‐Cy or PS‐Cy, with or without cisplatin pretreatment. (E) Quantitative analysis of fluorescence intensities corresponding to (D), (*n* = 3). (F) In vivo NIR fluorescence images of AKI model mice at different time points after intravenous injection of CyOH‐loaded PAMAM, PS1, PS2, and PS3. (G) Fluorescence intensities of mouse kidneys corresponding to (F), (*n* = 3). (H) Representative ex vivo fluorescence images of major organs harvested from AKI model mice at 2 h post‐intravenous injection of CyOH‐loaded PAMAM, PS1, PS2, and PS3. (I) Quantitative analysis of fluorescence intensities in major organs (*n* = 3). (J) In vivo NIR fluorescence images of healthy and AKI model mice after intravenous injection of CyOH‐loaded PS1. (K) Fluorescence intensities of mouse kidneys corresponding to (J), (*n* = 3). Data are presented as the mean ± SD; *n* = 3. An unpaired two‐tailed t‐test was used in panels (E and K), and one‐way ANOVA was used in panels (G and I). ns *p* > 0.05, * *p* < 0.05; ** *p* < 0.01; *** *p* < 0.001.

Cisplatin has been widely reported to induce the upregulated expression of KIM‐1 within renal tubular epithelial cells [53, 54]. To investigate the enhanced uptake of PS by injured renal cells, HK‐2 cells that had been pretreated with or without cisplatin stimulation were incubated with CyOH‐loaded PS (PS‐Cy) for 4 h. Compared with non‐cisplatin‐treated cells, cisplatin‐exposed cells exhibited significantly increased fluorescence intensity, which directly indicates enhanced cellular uptake of PS‐Cy (Figure 2D,E). To validate the role of *L*‐serine modification in KIM‐1‐mediated targeted delivery, a control probe (designated PAMAM‐Cy) was prepared, consisting exclusively of CyOH encapsulated within unmodified PAMAM dendrimers, and this control probe was co‐incubated with both healthy and cisplatin‐injured HK‐2 cells. Fluorescence imaging revealed that cisplatin‐exposed cells treated with PAMAM‐Cy displayed markedly weaker intracellular fluorescence compared to those treated with PS‐Cy. Confirmation of this specificity was obtained via a KIM‐1 antibody blocking assay. Preincubation of injured HK‐2 cells with KIM‐1 antibodies to shield cell‐surface epitopes significantly inhibited PS uptake (Figure S24A,B). Collectively, these findings confirm that KIM‐1 upregulation in cisplatin‐injured renal cells facilitates the selective internalization of PS‐Cy via specific interactions between KIM‐1 and the *L*‐serine moieties displayed on the surface of PS conjugates.

Subsequently, to systematically evaluate the targeting capability of PS conjugates toward injured renal tissues, a series of PS conjugates with varying *L*‐ser grafting ratios (38.6%, 57.2% and 85.8%) were synthesized (Figure S25), and their renal accumulation profiles were characterized via non‐invasive in vivo fluorescence imaging. Dorsal whole‐body fluorescence imaging analyses revealed marked differences in fluorescence signal intensity among PS variants with different serine grafting ratios in cisplatin‐induced AKI mouse models (Figure 2F,G). At 1 h post‐intravenous probe injection, all PS conjugates produced detectable fluorescence signals in the renal region of AKI mice, confirming their ability to selectively accumulate in the injured kidney tissue. Notably, the PS3 group (85.8% *L*‐ser grafting ratios) demonstrated significantly higher fluorescence intensity compared to all other PS variants 2 h after injection, indicating that this specific serine grafting ratio yields optimal renal targeting and delivery efficiency. Furthermore, ex vivo fluorescence imaging of the isolated kidneys further corroborated this key finding, as the fluorescence intensity in the renal tissue of the PS3 group was 3.7‐fold, 3.6‐fold, and 1.9‐fold higher than that of the PAMAM, PS2, and PS1 groups, respectively (Figure 2H,I). To further verify the injury‐specific targeting capability of PS3, its renal distribution was compared between healthy and AKI mice. The results revealed that the fluorescence intensity in the kidneys of AKI mice was approximately 3.2‐fold greater than that observed in healthy mice 2 h after injection (Figure 2J,K). This pronounced disparity in renal fluorescence accumulation is likely attributable to cisplatin‐induced upregulation of KIM‐1 in the injured renal tissue, as KIM‐1 specifically interacts with the serine moiety of PS to promote probe internalisation and retention. Taken together, these in vivo and ex vivo results indicate that the PS3 probe exhibits superior renal targeting specificity and extended tissue retention within the injured kidneys. Consequently, this optimised conjugate constitutes a robust and promising delivery platform for developing AKI‐targeted diagnostic probes.

### 2.4 In Vitro Cellular Imaging of H_2_O_2_ in Cisplatin‑Induced AKI Model

For the evaluation of RCy, DRCy, and QRCy in detecting H_2_O_2_ in the cellular environment, these three probes were separately encapsulated into PS3 nanocarriers to construct the composite nanoprobes PS‐RC, PS‐DRC, and PS‐QRC, respectively (Figure 3A). We quantitatively determined the drug loading and encapsulation efficiency of PS‐QRC to be 6.95% and 68.07%, respectively. The PS‐QRC exhibited satisfactory dispersibility and stability in PBS or PBS containing 10% fetal bovine serum (FBS). Despite 36 h of incubation, their diameters and polydispersity index (PDI) did not change significantly (Figure S26A,B). Furthermore, the fluorescence intensity of the PS‐QRC in PBS containing 10% FBS were highly consistent with those measured in PBS buffer (Figure S26C). Collectively, the nanoprobes display outstanding colloidal and optical stability under serum‐simulated blood conditions.

**FIGURE 3:**
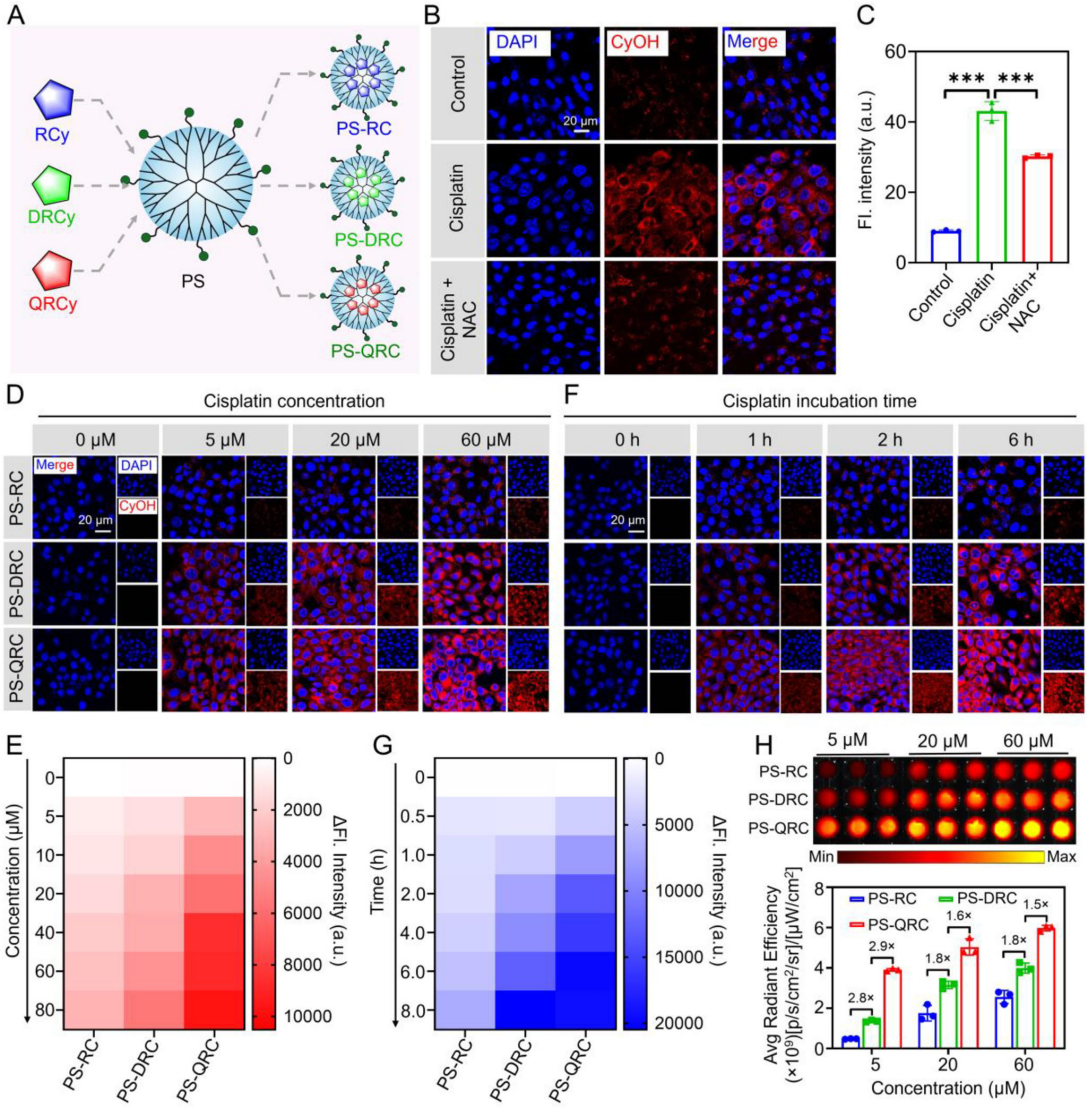
In vitro cellular imaging of H_2_O_2_ in a cisplatin‑induced AKI model. (A) Schematic diagram of the preparation of nanoprobes PS‐RC, PS‐DRC, and PS‐QRC. (B) Confocal fluorescence imaging of HK‐2 cells treated with cisplatin or pre‐treated with NAC for 2 h before cisplatin for 24 h, then incubated with PS‐QRC for 1 h. (C) Quantitative analysis of fluorescence intensities corresponding to (B), (*n* = 3). (D‐E) Confocal fluorescence imaging and quantitative analysis of HK‐2 cells treated with different concentrations of cisplatin for 6 h, then incubated with PS‐RC, PS‐DRC, and PS‐QRC for 1 h. (F‐G) Confocal fluorescence imaging and quantitative analysis of HK‐2 cells treated with different time of cisplatin, then incubated with PS‐RC, PS‐DRC, and PS‐QRC for 1 h. (H) Fluorescence images and intensity analysis of PS‐RC, PS‐DRC, and PS‐QRC after being treated with various cisplatin concentrations (*n* = 3). Data are presented as the mean ± SD; *n* = 3. Statistical significance was analyzed using one‐way ANOVA. *** *p* < 0.001.

Prior to in vitro fluorescence imaging investigations, the biocompatibility of all prepared nanoprobes was systematically evaluated. Cell viability assays corroborated that none of the nanoprobes elicited significant cytotoxicity or cell death following prolonged incubation with renal tubular epithelial cells (HK‐2), mouse embryonic fibroblast cells (3T3), and human umbilical vein endothelial cells (HUVECs) (Figure S27A–C). Furthermore, parallel assessments using the 2',7'‐Dichlorodihydrofluorescein diacetate fluorescent probe verified that none of the formulated nanoprobes induced an aberrant elevation in intracellular ROS levels (Figure S27D). This rigorous validation ensures that subsequent fluorescence changes were solely attributed to cisplatin‐induced oxidative stress rather than probe toxicity.

To further verify whether the nanoprobe can be employed for imaging drug‐induced cell injury, HK‐2 cells were treated with cisplatin for 6 h and then incubated with PS‐QRC for NIR fluorescence imaging. In the control group, HK‐2 cells were pretreated with *N*‐acetylcysteine (NAC, a well‐recognized ROS scavenger) for 2 h prior to cisplatin treatment. After 1 h of incubation, the NIR fluorescence intensity in cisplatin‐treated HK‐2 cells was significantly enhanced by 4.8‐fold (Figure 3B,C), whereas NAC pretreatment markedly attenuated the fluorescence increase. These results validate the feasibility of PS‐QRC for imaging drug‐induced cell injury.

Subsequently, we further evaluated the sensitivity of these self‐amplified probes in the detection of cisplatin‐induced damage. As the extent of cellular injury is directly correlated with the concentration and duration of drug exposure, we first examined the effects of different drug concentrations on the fluorescent response of the nanoprobes. HK‐2 cells were treated with gradient concentrations of cisplatin for 5 h and subsequently incubated with each probe for 1 h. As depicted in Figure 3D, negligible red fluorescence was observed in all probe groups in the absence of cisplatin stimulation, confirming a low background signal. With increasing cisplatin concentrations, the red fluorescence intensity of all probes increased gradually, consistent with the dose‐dependent elevation of intracellular H_2_O_2_ levels triggered by cisplatin‐induced oxidative stress. Notably, PS‐QRC displayed the strongest fluorescence signal at all cisplatin concentrations, and a clear and discernible signal was observed even at 5 µM cisplatin, whereas PS‐RC exhibited only faint activation. Quantitative fluorescence analysis further corroborated this trend (Figure 3E). In the time‐course assay, cells were treated with 60 µM cisplatin for varied time intervals and then incubated with the respective probes. As shown in Figure 3F,G, the fluorescence intensity of all probes increased progressively within 1–6 h, reflecting the time‐dependent accumulation of H_2_O_2_ in injured renal tubular cells. PS‐QRC again exhibited superior sensitivity, with a distinct fluorescent signal detectable as early as 1 h, while PS‐RC required a longer stimulation period to generate a measurable response. Taken together, these results underscore the superior sensitivity and responsiveness of PS‐QRC for real‑time imaging of oxidative stress dynamics associated with drug‑induced cell injury.

We further assessed the imaging performance of these probes toward drug‐induced cellular injury using the IVIS spectrum. The results were consistent with the aforementioned fluorescence imaging observations (Figure 3H). The fluorescence signals of all three probes increased gradually with elevated cisplatin concentrations and displayed an obvious dose‐dependent activation behavior. Even at the lowest cisplatin concentration of 5 µm, PS‐QRC exhibited a pronounced fluorescence signal, whereas PS‐RC generated only a weak response. These findings visually and quantitatively confirm that PS‐QRC displays markedly higher sensitivity toward low levels of ROS. Collectively, these cellular results fully demonstrate that PS‐QRC, as a self‐amplifying fluorescent probe featuring a multi‐fluorophore self‐immolative design, exhibits superior sensing performance relative to PS‐RC and PS‐DRC. Accordingly, this outstanding signal amplification capability endows PS‐QRC with great potential for the early optical diagnosis of AKI.

### 2.5 NIR Fluorescence Imaging of AKI Mice In Vivo

Encouraged by the excellent performance of self‐amplifying nanoprobes in imaging drug‐induced cellular injury, we further investigated the potential of PS‐RC, PS‐DRC, and PS‐QRC for real‐time fluorescence imaging of cisplatin‐induced AKI in vivo. Pharmacokinetic profiling revealed a short blood half‐life (t_1/2_ = 0.67 h) for RP‐SC in healthy mice, facilitating rapid systemic clearance (Figure S28A). Crucially, quantitative biodistribution analysis showed that 57.9% of RP‐SC accumulated in the kidneys of AKI mice at 2 h post‐injection, confirming effective renal targeting (Figure S28B). Building on this favorable pharmacokinetic profile, we established an AKI model via intraperitoneal cisplatin injection (20 mg/kg) and administered the nanoprobes intravenously at designated time points. Whole‐body fluorescence imaging was then performed from the back using the IVIS spectrum 2 h after the final injection (Figure 4A). Figure 4B showed that the kidney fluorescence increased as the cisplatin treatment time extended. Notably, at 12 h post‐cisplatin treatment, PS‐QRC exhibited a readily detectable fluorescent signal in the kidney region, whereas PS‐DRC and PS‐RC remained undetectable at this time point. Quantitative analysis (Figure 4C) revealed that the SBR achieved with PS‐QRC was 3.4‐fold higher than those of the other two groups. Between 12 and 24 h, the fluorescence signals of all nanoprobes increased progressively, with PS‐QRC displaying the most intense signal at 24 h. This time‐dependent activation pattern directly mirrors the progression of cisplatin‐induced oxidative stress and renal injury, with PS‐QRC clearly demonstrating the earliest activation and strongest signal intensity among the three probes. As shown in Figure 4D,E, ex vivo imaging of mouse organs showed that the kidneys of mice in the PS‐QRC group exhibited significantly enhanced fluorescence signals after 12 h of cisplatin injection, which were 2‐fold and 3.6‐fold higher than those in the PS‐DRC and PS‐RC groups, respectively. Furthermore, fluorescence imaging of renal slices at 2 h post‐injection revealed that these activated signals localized predominantly to the renal cortex rather than the medulla (Figure S29). This spatial confinement correlates with the high expression of KIM‐1 and elevated ROS levels characteristic of proximal tubular epithelial cells residing in the cortical region.

**FIGURE 4:**
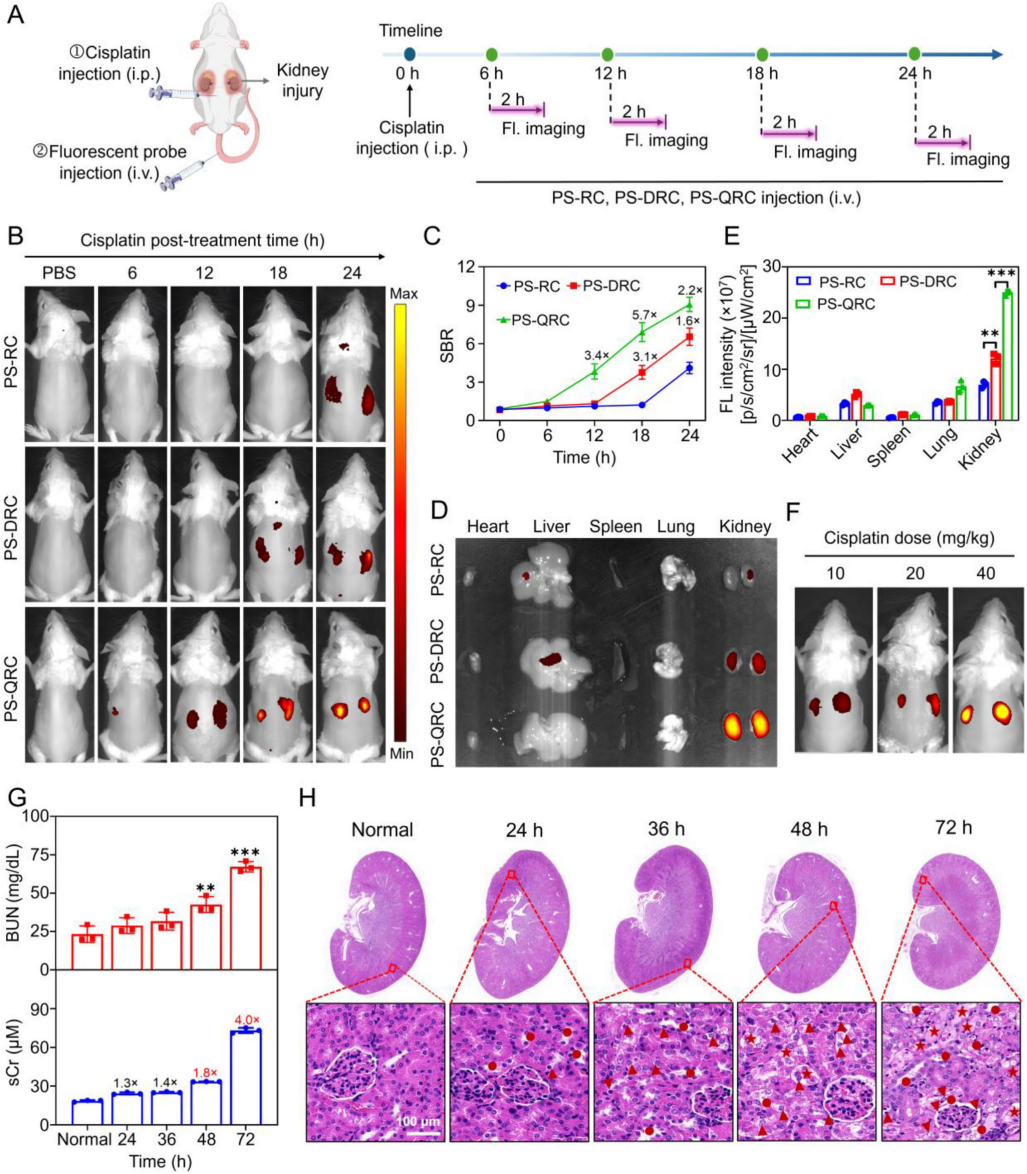
In vivo NIR fluorescence imaging of AKI mice. (A) Schematic illustration of the experimental protocol. (B) In vivo NIR fluorescence images of mice after intraperitoneal injection of cisplatin for different durations, followed by intravenous injection of PS‐RC, PS‐DRC, and PS‐QRC nanoprobes for 2 h. (C) Calculated SBR values corresponding to (B), (*n* = 3). (D) Representative ex vivo fluorescence images of major organs harvested from mice after intraperitoneal injection of cisplatin for 12 h and subsequent intravenous injection of PS‐RC, PS‐DRC, and PS‐QRC nanoprobes for 2 h. (E) Quantitative analysis of fluorescence intensities in major organs (*n* = 3). (F) In vivo NIR fluorescence images of mice after intraperitoneal injection of different doses of cisplatin for 12 h, followed by intravenous injection of RP‐SC for 2 h. (G) The levels of BUN and sCr in serum after injection of cisplatin for different time (*n* = 3). (H) H&E staining images of kidney tissue sections harvested from mice after treatment with cisplatin for different durations. Data are presented as the mean ± SD; *n* = 3. Statistical significance was analyzed using one‐way ANOVA. ** *p* < 0.01; *** *p* < 0.001.

After validating the excellent performance of PS‐QRC in accurately diagnosing AKI, we applied it for detecting the degree of AKI in vivo. A series of doses (10, 20, and 40 mg/kg) of cisplatin were intraperitoneally injected into mice to induce varying degrees of AKI, and PS‐QRC was intravenously injected for imaging. As presented in Figure 4F, the kidney regions of AKI mice displayed strong fluorescence signals, whose intensity gradually increased with elevated cisplatin dosage. Specifically, in AKI induced by 40 mg/kg cisplatin, the SBR reached 5.7, which was markedly higher than 3.6 at 20 mg/kg and 2.4 at 10 mg/kg (Figure S30). These results demonstrate that PS‐QRC can be employed to evaluate the severity of AKI at different stages. Importantly, even in mice with mild AKI, the imaging performance of PS‐QRC was significantly superior to that of PS‐DRC and PS‐RC, enabling earlier detection of elevated ROS levels in injured kidneys (Figure S31A,B). Collectively, these findings underscore the distinct advantages of this signal‐amplification activatable probe for the early diagnosis of AKI.

Furthermore, in the cisplatin‐induced AKI model, hematoxylin and eosin (H&E) staining revealed no obvious acute tubular injury within 24 h post‐treatment (Figure 4H). In contrast, the loss of brush borders, tubular dilation, intraluminal cellular debris, and hyaline casts were evidently observed in the 48‐h group. Biochemical analysis of renal function demonstrated that the BUN level was significantly elevated compared with the normal group only at 48 h post‐cisplatin injection, whereas the sCr level reached 1.8 times that of the control (Figure 4G). In accordance with the KDIGO 2026 Clinical Practice Guideline for Acute Kidney Injury (AKI) and Acute Kidney Disease (AKD), a ≥1.5‑fold rise in sCr from baseline serves as a core diagnostic threshold for AKI. These results confirmed that PS‐QRC‐based fluorescence imaging enables earlier warning of early AKI occurrence compared with traditional blood biomarkers and tissue H&E staining, thereby establishing PS‐QRC as a reliable tool for the precise diagnosis of early‐stage AKI. Additionally, blood biochemistry analysis confirmed that PS‐QRC induced no abnormalities in liver and kidney function (Figure S32A–D). H&E staining results of various organs also indicated no significant side effects of PS‐QRC (Figure S33), collectively demonstrating its excellent biocompatibility.

### 2.6 High‐Throughput Screening of Natural Products

Early diagnosis of AKI is critical for timely intervention to halt disease progression and reduce related mortality. The current absence of targeted therapeutic strategies for AKI highlights the demand for effective therapeutic agents. Encouraged by the excellent performance of PS‐QRC in live‐cell and in vivo imaging, we further investigated its potential as a reliable screening tool for antioxidants that can reduce ROS production. Accordingly, we established a simple and efficient high‐throughput screening platform based on PS‐QRC to discover natural products with preventive or therapeutic efficacy against AKI. As illustrated in Figure 5A, for therapeutic screening, HK‐2 cells were first treated with 60 µM cisplatin for 5 h and then incubated with 50 µM natural compounds for another 12 h. For prophylactic screening, cells were pretreated with 50 µM natural products for 12 h before exposure to 60 µM cisplatin for 5 h. After these treatments, cells were incubated with PS‐QRC for 1 h and then subjected to fluorescence imaging to measure intracellular ROS levels. This experimental strategy supports the rapid and effective identification of natural products that can alleviate oxidative stress related to AKI.

**FIGURE 5:**
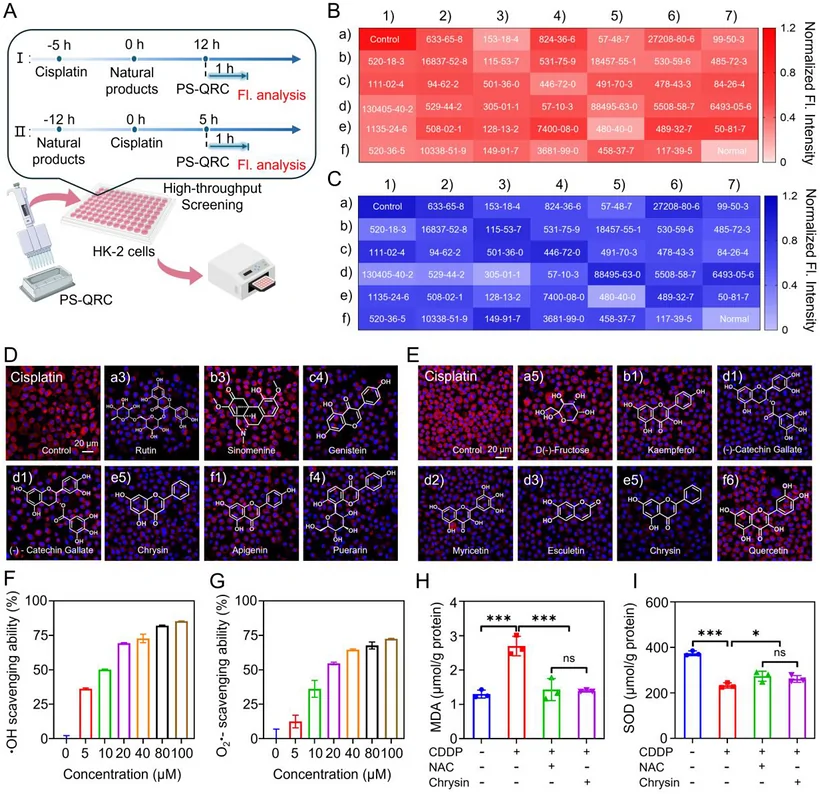
High‑throughput screening of antioxidant natural products in living HK‑2 cells toward AKI treatment. (A) Schematic illustration of the high‑throughput screening strategy for antioxidant natural products using a microplate reader. (B) Normalized fluorescence intensity of HK‑2 cells treated with different natural products after cisplatin‑induced injury, followed by incubation with RS‑QRC for 1 h. (C) Normalized fluorescence intensity of HK‐2 cells pretreated with different natural products prior to cisplatin challenge, followed by incubation with RS‐QRC for 1 h. (D) Confocal fluorescence images of HK‐2 cells treated with different natural products after cisplatin‐induced injury, followed by incubation with RS‐QRC for 2 h. (E) Confocal fluorescence images of HK‐2 cells pretreated with different natural products prior to cisplatin challenge, followed by incubation with RS‐QRC for 2 h. (F) ·OH scavenging capacity of chrysin at different concentrations (*n* = 3). (G) O_2_·^−^ scavenging capacity of chrysin at different concentrations (*n* = 3). (H) SOD activities in HK‐2 cells following various treatments (*n* = 3). (I) MDA levels in HK‐2 cells following various treatments (*n* = 3). Data are presented as the mean ± SD; *n* = 3. Statistical significance was analyzed using one‐way ANOVA. ns *p* > 0.05, * *p* < 0.05, ** *p* < 0.01, *** *p* < 0.001.

With this established screening platform, we screened 40 natural compounds and evaluated their effects on intracellular ROS production. As shown in Figure 5B, compared with the cisplatin only control, cells in the cisplatin‐pretreated group treated with compounds a3 (rutin), b3 (sinomenine), c4 (genistein), d1 ((‐)‐epicatechin gallate), e5 (chrysin), f1 (apigenin), and f4 (puerarin) exhibited attenuated fluorescence intensity at 700 nm. In the natural compound pretreated group, compounds a5 (D(‐)‐fructose), b1 (kaempferol), d1 ((‐)‐catechin gallate), d2 (myricetin), d3 (esculetin), e5 (chrysin), and f6 (quercetin) also elicited reduced fluorescence signals, which indicates potent ROS‐scavenging activity (Figure 5C). These results were further confirmed by confocal microscopic imaging (Figure 5D,E). The cisplatin only control displayed intense red fluorescence, indicating high levels of ROS accumulation in injured renal tubular cells. In contrast, treatment with the screened natural compounds obviously decreased intracellular red fluorescence intensity, which confirms their ability to limit ROS production or remove excess ROS.

Notably, chrysin (compound e5), a natural flavonoid abundant in *P* *assiflora caerulea* and honeybee products, emerged as the most promising candidate. Our results reveal that chrysin functions dually as both a prophylactic agent and a therapeutic agent. It effectively reduces cisplatin triggered ROS elevation when administered before cisplatin exposure and alleviates ROS accumulation when delivered after cisplatin treatment. We further explored the cytoprotective mechanisms of chrysin and found that it directly scavenges hydroxyl radicals and superoxide anions with 69.2% and 54.6% scavenging efficiencies at 20 µM, respectively (Figure 5F,G). Cell experiments further confirmed that chrysin significantly reduced cisplatin induced increase in intracellular malondialdehyde (MDA) as shown in Figure 5H. The efficacy is similar to that of the classic antioxidant NAC. Chrysin also improved superoxide dismutase (SOD) activity in injured cells (Figure 5I). Collectively, these results demonstrate that chrysin protects against renal injury through a dual mechanism. It acts by direct ROS scavenging and by activating the endogenous antioxidant defense system. These properties support its ability to reduce excessive ROS production in renal cells exposed to nephrotoxic agents and support its role as a promising lead compound for AKI treatment. These findings confirm that PS‐QRC represents a valuable high throughput screening platform. It provides a reliable and effective strategy for identifying and prioritizing natural products with renoprotective potential for AKI.

### 2.7 Monitoring of Therapeutic Effect on AKI

To assess the in vivo renoprotective efficacy of chrysin and verify whether PS‐QRC can reliably monitor therapeutic outcomes in live animals, we utilized a cisplatin‐induced AKI mouse model, with chrysin administered as the test therapeutic agent and NAC serving as a positive control, and treatment effects were evaluated by monitoring the fluorescence variations of PS‐QRC. As depicted in Figure 6A, healthy BALB/c mice were given intraperitoneal injections of cisplatin at a nephrotoxic dose of 20 mg/kg to establish AKI, then treated with chrysin or NAC at a single dose (1×, administered 12 h post‐cisplatin), double dosages (2×, administered 12 and 24 h post‐cisplatin), and triple dosages (3×, administered 12, 24, and 36 h post‐cisplatin), respectively. Mice in the control group were treated with an equivalent volume of saline following cisplatin administration. Renal ROS levels were quantified via PS‐QRC fluorescence imaging 12 h after the final treatment dose. As presented in Figure 6B, fluorescence imaging revealed a dose‐dependent reduction in renal fluorescence intensity in chrysin‐treated mice compared with the cisplatin‐only control group, confirming progressive mitigation of renal oxidative stress with increasing chrysin dosages. Corresponding quantitative analysis further demonstrated that the SBR in renal decreased from 12.3 to 7 following single‐dose chrysin treatment, to 5.3 after double‐dose chrysin administration, and to 3 upon triple‐dose chrysin treatment (Figure 6C). A similar trend was observed in the kidneys of NAC‐treated mice, indicating that the efficacy of chrysin in alleviating cisplatin‐induced renal oxidative stress is comparable to that of NAC. Furthermore, subsequent fluorescence analysis corroborated that pretreatment with chrysin effectively attenuated cisplatin‐induced renal oxidative stress in mice (Figure 6D,E). Taken together, these in vivo imaging findings directly validate that chrysin effectively mitigates cisplatin‐triggered renal oxidative stress in a significant dose‐dependent manner.

**FIGURE 6:**
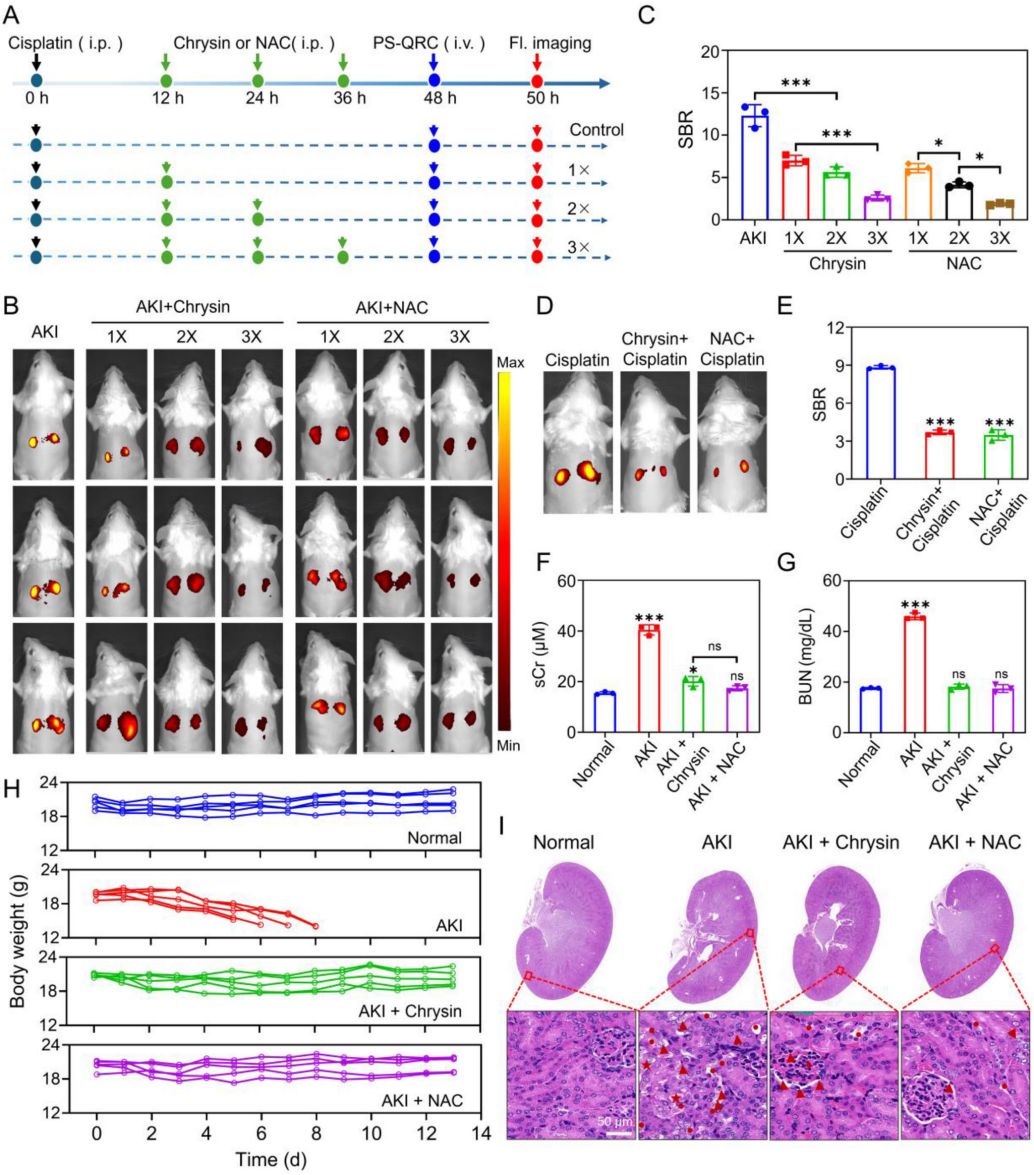
In vivo monitoring AKI therapeutic effects. (A) Schematic illustration of the experimental protocol. (B) In vivo fluorescence images of AKI mice after intraperitoneal injection of different doses of chrysin and NAC, followed by intravenous injection of RS‐QRC for 2 h. (C) Calculated SBR values corresponding to (B), (*n* = 3). (D) In vivo fluorescence images of mice with intraperitoneal injection of chrysin and NAC prior to cisplatin challenge, followed by intravenous injection of RS‐QRC for 2 h. (E) Calculated SBR values corresponding to (D), (*n* = 3). The levels of sCr (F) and BUN (G) in different treatment groups (*n* = 3). (H) Body weight change of mice throughout the treatment period (*n* = 5). (I) H&E staining images of kidney tissue sections harvested from mice after different treatments. Data are presented as the mean ± SD; *n* = 3 – 5. Statistical significance was analyzed using one‐way ANOVA. ns *p* > 0.05, * *p* < 0.05; ** *p* < 0.01; *** *p* < 0.001.

To verify the reliability of PS‐QRC as a probe for therapeutic efficacy evaluation, we further monitored mouse body weight, survival rate and serum renal function biomarkers (sCr and BUN). As illustrated in Figure 6H, mice treated with chrysin or NAC maintained steady body weight throughout the entire observation period, whereas the cisplatin‐only control group displayed pronounced and progressive body weight loss. Survival analysis presented in Figure S34 further demonstrated that both chrysin and NAC treatment completely reversed cisplatin‐induced mortality, elevating the survival rate from 0% in the cisplatin‐only group to 100% in both treatment groups. Regarding serum renal function markers, chrysin treatment reduced sCr levels by twofold and BUN levels by 2.5‐fold, respectively, compared with the untreated AKI group, and these levels gradually approached the normal baseline of healthy mice (Figure 6F,G). Similarly, NAC administration exerted nearly equivalent ameliorating effects on sCr and BUN levels, confirming that the renoprotective efficacy of chrysin is comparable to this classic clinical antioxidant. H&E staining of mouse renal tissue further verified that chrysin and NAC both effectively alleviated cisplatin‐induced renal tubular cell injury (Figure 6I). These results were highly consistent with the fluorescence imaging outcomes obtained using the PS‐QRC probe, further confirming that chrysin treatment effectively restored renal function in cisplatin‐induced AKI mice. Collectively, these findings indicate that the designed PS‐QRC probe enables efficient, real‐time monitoring of the therapeutic effects of chrysin and NAC against AKI, and holds considerable potential for facilitating the development and optimisation of novel therapeutic strategies for AKI through real‐time fluorescence imaging.

## 3 Conclusions

In this study, we successfully developed a kidney‐targeted, self‐amplifying NIR fluorescent nanoprobe (PS‐QRC) for the early diagnosis of AKI and high‐throughput screening of natural kidney‐protective compounds. Both in vitro and in vivo experiments demonstrated that PS‐QRC specifically reacts to H_2_O_2_ by self‐fragmenting to release multiple fluorescent molecules, enabling inherent signal amplification and extremely sensitivity for early AKI detection. Additionally, modification with *L*‐serine facilitates specific binding to KIM‐1, enhancing targeted accumulation in the kidneys and minimizing background noise. PS‐QRC also allows for noninvasive, real‐time imaging of AKI as early as 12 h, offering earlier detection compared to conventional serum biochemical and histopathological techniques, and it can continuously monitor the progression and severity of AKI. Moreover, this nanoprobe functions as a powerful high‐throughput screening platform, identifying chrysin as a promising kidney‐protective agent with effectiveness similar to NAC, while enabling real‐time quantitative tracking of its dose‐dependent therapeutic effects. In summary, this self‐amplifying activatable fluorescent nanoprobe not only provides sensitive early diagnosis and dynamic treatment monitoring of AKI but also serves as a dependable tool for discovering new kidney‐protective drugs.

## 4 Methods

### 4.1 Preparation and Characterization of PS‐RC, PS‐DRC, and PS‐QRC Nanoprobes

In brief, 10 mg of PS1 (see Supporting Information for details) was dissolved in 5 mL of ultrapure water. A solution of 1 mg of the probes (PS‐RC, PS‐DRC, or PS‐QRC) dissolved in 0.5 mL of DMSO was added dropwise into the PS1 solution while stirring at 1000 rpm for 12 h. The mixture was transferred into a dialysis bag (MWCO = 3500) and dialyzed against ultrapure water for 24 h to remove DMSO, and the mixture was filtered through a 0.22 µM filter to obtain nanoprobes. The particle size and morphology were studied by using DLS and TEM. The probes loading efficiency was studied by using a UV‐3802 spectrophotometer.

### 4.2 Determination of the Responsiveness Toward H_2_O_2_

To evaluate the sensitivity of RCy, DRCy, and QRCy toward H_2_O_2_, the compounds (5 µM each) were incubated with varying concentrations of H_2_O_2_ (0, 0.1, 0.5, 1, 5, 10, 20, 40, 80 µm) in buffer at 37°C for 2 h. The fluorescence spectra were recorded using a fluorescence spectrophotometer with an excitation wavelength of 660 nm. The resulting fluorescence intensities at 700 nm were plotted against the concentrations of H_2_O_2_, and a linear regression was fitted to obtain the slope k. The detection limit was calculated using the formula 3σ/k, where σ represents the standard deviation of six blank measurements. To further investigate the specificity toward H_2_O_2_, the compounds (5 µM each) were incubated with H_2_O_2_, GSH, Cys, Arg, Ca^2+^, Fe^3+^, Cu^2+^, Na^+^, Cl^−^ or NO_2_^−^ at 37°C for 2 h. The fluorescence intensity at 700 nm was recorded on multi‐functional microporous plate analysis system at an excitation of 660 nm.

### 4.3 Cell Culture

Human proximal renal tubular epithelial HK‐2 cells were obtained from Jiangsu Keygen Biotech Corp., Ltd (Nanjing, China). HK‐2 cells were cultured in Dulbecco's Modified Eagle's Medium/Ham's Nutrient Mixture F12 (DMEM/F12) supplemented with 10% fetal bovine serum (FBS), 100 µg/mL streptomycin, and 100 U/mL penicillin. The cells were maintained in a 100% humidified atmosphere containing 5% CO_2_ at 37°C. The culture medium was refreshed daily, and the cells were subcultured upon reaching confluence.

### 4.4 Cytotoxicity Evaluation

The cytotoxicity of PS‐RC, PS‐DRC, and PS‐QRC on HK‐2 cells was evaluated by a standard Cell Counting Kit‐8 (CCK‐8) assay. In brief, HK‐2 cells were seeded in 96‐well plates at an initial density of 5000 cells per well in 100 µL of complete medium. After incubation at 37°C for 24 h, the medium was removed, and fresh medium containing different concentrations of PS‐RC, PS‐DRC, and PS‐QRC was added to each well, followed by incubation for another 24 h. The medium containing the tested compounds was then removed, and 100 µL of medium supplemented with 10 µL of CCK‐8 reagent was added to each well, followed by incubation for 1 h. The absorbance of each well at 450 nm was measured using a microplate reader to calculate the cell viability.

### 4.5 Effects of Nanoprobes on Intracellular ROS Levels in HK‐2 Cells

The intracellular ROS levels in HK‐2 cells treated with PS‐RC, PS‐DRC, and PS‐QRC were evaluated using the commercial ROS probe dichlorodihydrofluorescein diacetate (DCFH‐DA). In brief, HK‐2 cells were seeded in 96‐well plates at an initial density of 5000 cells per well in 100 µL of complete medium. After incubation at 37°C for 24 h, the medium was removed, and fresh medium containing different concentrations of PS‐RC, PS‐DRC, and PS‐QRC was added to each well, followed by incubation for another 24 h. The medium containing the tested compounds was then removed, and 100 µL of medium supplemented with 0.1 µL of DCFH‐DA was added to each well, followed by incubation for 30 min. The fluorescence intensity of each well was measured using a spectrofluorometer at an excitation wavelength (λ_ex_) of 480 nm and an emission wavelength (λ_em_) of 525 nm.

### 4.6 Detection of PS's Targeting Ability to Cisplatin‐Induced Kidney Cell Injury

HK‐2 cells were seeded into 35‐mm confocal glass‐bottom dishes at a density of 1 × 10^4^ cells per dish and incubated in complete medium at 37°C overnight. After stimulation with or without cisplatin (50 µM) for 12 h, the HK‐2 cells were treated with CyOH‐loaded PS (PS‐Cy) at a concentration of 5 µM for an additional 4 h. The cells were observed using a confocal laser scanning microscope (Zeiss, LSM800). Fluorescence intensity was quantitatively analyzed using ImageJ software. CyOH‐loaded PAMAM (PAMAM‐Cy) was also tested as a control.

### 4.7 Detection of H_2_O_2_ Levels in HK‐2 Cells

HK‐2 cells were seeded into 35‐mm confocal dishes at a density of 1 × 10^4^ cells per dish and incubated in complete medium at 37°C. After 24 h, the medium was replaced with fresh serum‐free culture medium containing different concentrations of cisplatin, followed by incubation at 37°C for 5 h. Alternatively, the cells were treated with 60 µM cisplatin and incubated for varying durations. The medium was then replaced with fresh culture medium containing PS‐RC, PS‐DRC, and PS‐QRC (5 µM each), and the cells were incubated at 37°C for 1 h. After washing the cells three times with PBS buffer to remove any surface‐adhered materials, the cells were observed using a confocal laser scanning microscope. For quantitative analysis, HK‐2 cells were seeded in 96‐well black plates at an initial density of 5000 cells per well in 100 µL of complete medium. After undergoing the same treatment procedure described above, the fluorescence intensity at 700 nm was recorded using a multifunctional microplate reader with an excitation wavelength of 660 nm.

### 4.8 High‐Throughput Screening of Natural Products

HK‐2 cells were seeded in 96‐well black plates at an initial density of 5000 cells per well and cultured overnight. For therapeutic screening, HK‐2 cells were first treated with 60 µM cisplatin for 5 h, followed by incubation with 50 µM natural compounds for an additional 12 h. For prophylactic screening, cells were pretreated with 50 µM natural products for 12 h prior to exposure to 60 µM cisplatin for 5 h. Following these treatments, fluorescence intensity at 700 nm was measured using a multifunctional microplate reader at an excitation wavelength of 660 nm.

### 4.9 Animal Model

Female BALB/c mice (6‐8 weeks old, weighing 17–20 g) were purchased from Zhuhai BesTest Bio‐Tech Co., Ltd and maintained under specific pathogen‐free (SPF) conditions in the animal facility of Jinan University. All animal procedures were approved by the Institutional Laboratory Animal Care and Use Committee of Jinan University (No. IACUC‐20250801‐09) and performed in accordance with the committee's guidelines. To establish an AKI mouse model, mice were intraperitoneally (i.p.) injected with cisplatin at a dose of 20 mg kg^−1^ body weight. Mice receiving an i.p. injection of 200 µL saline were served as the control group. Animals were immediately sacrificed when the body weight loss exceded 20%.

### 4.10 Detection of PS's Targeting Ability to Kidney In Vivo

AKI mice were randomly divided into four groups with three mice per group. PAMAM‐Cy, PS1‐Cy, PS2‐Cy, and PS3‐Cy were administered via tail vein injection. Whole‐body fluorescence distribution was observed at 1 and 2 h post‐injection. Two hours after injection, the mice were sacrificed, and major organs were harvested for ex vivo fluorescence analysis. To verify the targeting ability of *L*‐ser modified nanocarriers to damaged renal cells, the distribution of PS3‐Cy in the kidneys of healthy mice and AKI mice was compared. The fluorescence signal intensities within the kidneys were quantitatively analyzed using region of interest (ROI) analysis.

### 4.11 In Vivo NIR Fluorescence Imaging in Living Mice

To evaluate the feasibility of using nanoprobes for in vivo kidney injury imaging, an AKI mouse model was established. Mice were subjected to intraperitoneal injection of cisplatin to induce renal damage. At specific time points (0, 6, 12, 18, and 24 h) post‐cisplatin administration, the mice were intravenously injected with nanoprobes (PS‐RC, PS‐DRC, or PS‐QRC). Two hours after nanoprobes injection, whole‐body NIR fluorescence imaging was performed. The fluorescence signal intensities within the kidneys were quantitatively analyzed using ROI analysis. The SBR was calculated by comparing the fluorescence intensity observed in the kidney region with that measured in the dorsal skin area. For ex vivo imaging, the mice were intravenously injected with PS‐RC, PS‐DRC, and PS‐QRC at 12 h post‐cisplatin injection. Two hours later, the mice were euthanized, and the major organs (heart, liver, spleen, lungs, and kidneys) were harvested for ex vivo fluorescence imaging. To induce varying degrees of kidney injury, different doses of cisplatin (10, 20, and 40 mg kg^−1^) were administered. Twelve hours post‐cisplatin injection, fluorescence imaging was performed on the mice using the nanoprobes.

### 4.12 Histological Analysis and Renal Function Assay

The kidneys were collected from sacrificed mice at different time points following cisplatin injection, fixed in 4% paraformaldehyde (PFA), embedded in paraffin, and sectioned at a thickness of 5 µM for H&E staining. Blood was collected by eyeball extraction at the time of sacrifice. The collected blood samples were allowed to stand at room temperature for 1 h and then centrifuged at 3000 r.p.m. for 15 min. BUN and sCr levels were analyzed using commercial kits, with 3 mice per group.

### 4.13 Evaluating the Therapeutic Effect of Chrysin on AKI

Healthy BALB/c mice were randomly selected and i.p. injected with cisplatin (20 mg kg^−1^) to induce AKI, followed by treatment with no chrysin (0×) or chrysin (50 mg/kg, i.p.) at a single dosage (1×, administered at 12 h), double dosages (2×, administered at 12 and 24 h), and triple dosages (3×, administered at 12, 24, and 36 h), respectively. NAC was used as positive control. At 48 h post‐cisplatin injection, PS‐QRC was intravenously (*i.v*.) injected, and whole‐body fluorescence images were acquired at 2 h post‐PS‐QRC injection. The study also examined changes in body weight and survival rate during the treatment period, the levels of BUN and sCr in mouse blood after three chrysin or NAC treatments, and H&E staining of kidney tissues to determine the therapeutic efficacy. In the kidney injury prevention experiment, healthy mice were first intraperitoneally injected with chrysin or NAC, and cisplatin was injected again 12 h later. Kidney fluorescence was detected using nanoprobes 24 h after cisplatin injection. The fluorescence signal intensities within the kidneys were quantitatively analyzed using ROI analysis.

### 4.14 Statistical Analysis

Data were presented as mean ± standard deviation (SD) in at least three independent experiments unless stated otherwise. Statistical comparisons between two groups were carried out using unpaired two‐tailed t‐test and multiple group comparisons were assessed using one‐way analysis of variance (ANOVA) for statistical analysis. ns = no statistical difference, *p < 0.05 was considered statistically significant, *p < 0.05, **p < 0.01, and ***p < 0.001.

## Author Contributions

Y.C., M.L., and M.D. contributed equally to this work. Y.Y. and K.W. conceived the idea. Y.C., C.Z., M.G., and Z.H. synthesized and characterized the materials. M.L., M.D., and Y.L. performed the studies on cells and animals. Y.C., Y.Y., W.X., Y. Z., and K.W. analyzed the data and wrote the paper.

## Conflicts of Interest

The authors declare no conflicts of interest.

## Supporting information




**Supporting File**: advs77162‐sup‐0001‐SuppMat.docx.

## Data Availability

The data that support the findings of this study are available from the corresponding author upon reasonable request.
