# Supplementary material for: Self‐Amplified Activatable Fluorescent Probe for Early Diagnosis of Acute Kidney Injury and High‐Throughput Screening of Natural Protectants
Source: Adv Sci (Weinh). 2026 Aug 13:e77162. Online ahead of print. doi: 10.1002/advs.77162 (PMC13472104; doi:10.1002/advs.77162)
Supplement: Supplementary file 1 [file ADVS-9999-e77162-s001.docx]

[Supporting Information for](https://www.sohu.com/a/406564779_120462441)

Self-Amplified Activatable Fluorescent Probe for Early Diagnosis of Acute Kidney Injury and High-Throughput Screening of Natural Protectants

*Yahui Chen, Meihong Liu, Mengchao Ding, Ye Liu, Chuanxu Zhu, Mingye Gong, Zhixuan Huang, Yi Zhang*, Wei Xue*, Youyong Yuan*, and* *Kewei Wang**

Y. Chen, M. Liu, C. Zhu, M. Gong, Z. Huang, Y. Zhang, W. Xue, K. Wang

Key Laboratory of Biomaterials of Guangdong Higher Education Institutes, Department of Biomedical Engineering and MOE Key Laboratory of Tumor Molecular Biology, Jinan University, Guangzhou, 510632, P. R. China

E-mail addresses: [zhangyi_0424hot@163.com](mailto:zhangyi_0424hot@163.com) (Y. Zhang), [weixue_jnu@aliyun.com](mailto:weixue_jnu@aliyun.com) (W. Xue), [wangkw@jnu.edu.cn](mailto:wangkw@jnu.edu.cn) (K. Wang).

M. Liu, K. Wang

Guangdong Provincial Key Laboratory of Spine and Spinal Cord Reconstruction, The Fifth Affiliated Hospital of Jinan University (Heyuan Shenhe People’s Hospital), Jinan University, Heyuan, 517000, P.R. China

M. Ding, Y. Liu, Y. Yuan

School of Biomedical Sciences and Engineering, Guangzhou International Campus, South China University of Technology, Guangzhou, 511442, P.R. China

E-mail addresses: [yuanyy@scut.edu.cn](mailto:yuanyy@scut.edu.cn) (Y. Yuan)

**KEYWORDS:** acute kidney injury, self-amplifying fluorescent sensor, self-immolative, high-throughput screening, chrysin

**Experimental section**

**Materials and characterization**

2,3,3-Trimethylindolenine, iodoethane, 4-hydroxybenzyl alcohol, 4-(bromomethyl) benzeneboronic acid pinacol ester, 4-nitrophenyl chloroformate, *N*,*N*'-dimethyl-1,2-ethanediamine, and tert-butyldimethylsilyl chloride were purchased from Energy Chemical (Shanghai, China). All other chemicals used in the experiments were purchased from Sigma-Aldrich Co., LLC. (Shanghai, China) and used without further purification. Dulbecco's Modified Eagle's Medium/Ham's Nutrient Mixture F12 (DMEM/F12), fetal bovine serum (FBS), streptomycin, and penicillin were purchased from Thermo (Shanghai, China).

^1^H NMR and ^13^C NMR spectra were measured on a Bruker ARX 400 NMR spectrometer (Bruker, Billerica, MA). Deuterated dimethyl sulfoxide (DMSO-*d*_6_), and deuterated chloroform (CDCl_3_) were used as the solvent for NMR measurements. The absorption spectra were measured on a UV-3802 (UNICO, Shanghai, China) spectrophotometer. Fluorescence spectra were measured on a HITACHI F-7100 spectrofluorometer (HITACHI, Japan). Particle size and size distribution were measured in an aqueous solution with a Malvern ZS90 dynamic light scattering instrument (Malvern Instruments Ltd., England) with a He-Ne laser (633 nm) and 90° collecting optics. The data were analyzed using Malvern Dispersion Technology Software 7.0.2. Absorbance and fluorescence intensity were determined by a multi-functional microporous plate analysis system (Tecan M200PRO, Switzerland). Confocal laser scanning microscopy (CLSM) images were taken with a Zeiss LSM 880 Confocal Microscope (Carl Zeiss, USA). *In vivo* and *ex vivo* fluorescence images of animals were acquired on the In Vivo Imaging System (IVIS, PerkinElmer, USA).

**Scheme S1.** Synthetic procedures of CyOH.

**Synthesis of Compound 1.** 2,3,3-trimethylindolenine (10 ml, 0.062 mol) and iodoethane (14.5 g, 0.093 mol) were mixed and heated under reflux for 24 h. The reaction mixture was cooled to room temperature and filtered. The precipitate was washed with hexane (3×10 mL) to afford pink crystals (15.2 g, yield 91.8%).

**Synthesis of Compound 2.** A mixture of dimethylformamide (20 mL) and methylene chloride (20 mL) was cooled in an ice bath. Phosphorus oxychloride (18 mL) dissolved in methylene chloride (15 mL) was added dropwise with stirring, followed by cyclohexanone (5 g). The solution was refluxed for 3 h, cooled, poured into 200 g of ice, and allowed to stand overnight. The resulting yellow solid was filtered and dried, and used directly in the next step without further purification.

**Synthesis of Compound 3.** Under a nitrogen atmosphere, Compound 1 (3.65 g, 11.6 mmol) and sodium acetate (0.95 g, 11.6 mmol) were added to a round‑bottom flask containing Compound 2 (1.00 g, 5.79 mmol) in 15 mL of acetic anhydride. The solution was heated at 130 °C for 1 h. The mixture was filtered, and the precipitate was washed thoroughly with diethyl ether and aqueous potassium iodide solution to give a dark green solid with metallic luster (3.35 g, 90.5%).

**Synthesis of CyOH.** Under a nitrogen atmosphere, K₂CO₃ (311 mg, 2.3 mmol) was added rapidly to a round‑bottom flask containing resorcinol (248 mg, 2.3 mmol) in 5 mL of anhydrous acetonitrile. The mixture was stirred at ambient temperature for 10 min. Compound 3 (575 mg, 0.9 mmol) in 5 mL of anhydrous acetonitrile was then added. The reaction was heated at 50 °C for 2 h. Upon completion, the solvent was evaporated, and the residue was purified by silica gel column chromatography using dichloromethane/methanol (100:1 to 10:1, v/v) as the eluent to afford the desired product as a blue powder (225 mg, yield 48%).

**Scheme S2.** Synthetic procedures of RCy.

**Synthesis of Compound 4.** 4-Hydroxybenzyl alcohol (2.0 g, 11.9 mmol) and K₂CO₃ (1.84 g, 27.0 mmol) were dissolved in acetone (50 mL), followed by addition of 4-bromomethylphenylboronic acid pinacol ester (3.45 g, 25 mmol). The reaction mixture was heated at 60 °C under reflux for 4 h. Upon completion, the organic solvent was removed, and the crude product was purified by column chromatography (petroleum ether/ethyl acetate = 20:1, v/v) to afford Compound 4 as a pale yellow oil (5.4 g, 82.2%).

**Synthesis of RCy.** Compound 4 (150 mg, 0.7 mmol) was dissolved in DCM. Et_3_N (0.1 mL, 0.77 mmol) and a catalytic amount of DMAP were added. The mixture was cooled to 0 °C, and a solution of PNP chloroformate (155.2 mg, 0.82 mmol) in 20 mL of DCM was added dropwise. The reaction was stirred at room temperature for 16 h. The solvent was removed under reduced pressure, and the residue was redissolved in DCM. A solution of CyOH (306.9 mg, 0.77 mmol) in 10 mL of DCM was then added dropwise at 0 °C. After the addition, the reaction mixture was warmed to room temperature and stirred overnight. Upon completion, the solvent was evaporated, and the residue was purified by silica gel column chromatography using dichloromethane/methanol (100:1 to 10:1, v/v) as the eluent to afford the desired product as a blue powder (202 mg, yield 34%).

**Scheme S3.** Synthetic procedures of DRCy.

**Synthesis of Compound 5.** 2,6-Bis-(hydroxymethyl)-p-cresol (2.0 g, 11.9 mmol) and imidazole (1.84 g, 27.0 mmol) were dissolved in anhydrous DMF (10 mL) in a 100 mL round-bottom flask at 0 ℃ with constant stirring. A solution of tert-butyldimethylsilyl chloride (3.93 g, 26.2 mmol) in anhydrous DMF (5 mL) was then added. The reaction mixture was stirred at room temperature for 2 h, Upon completion, the mixture was washed with water and extracted with dichloromethane (3×50 mL). The organic layer was dried over anhydrous MgSO_4_, and the solvent was evaporated under reduced pressure. The crude product was purified by column chromatography to afford Compound 5 as a colorless oil (4.25 g, 90.2%).

**Synthesis of Compound 6.** Compound 5 (4.25 g, 10.7 mmol) and anhydrous potassium carbonate (1.85 g, 13.4 mmol) was dissolved in anhydrous acetonitrile (10 mL) in a 50 mL round-bottom flask at 0 ℃ with constant stirring for 10 min. 4-Bromomethylphenylboronic acid pinacol ester (3.17 g, 10.7 mmol) was added, and the mixture was stirred at room temperature overnight. Upon completion, the solvent was removed, and the crude product was purified by column chromatography (petroleum ether/ethyl acetate = 20:1, v/v) to afford Compound 6 as a pale-yellow oil (5.4 g, 82.2%).

**Synthesis of Compound 7.** Compound 6 (3 g, 4.8 mmol) was dissolved in tetrahydrofuran (10 mL) in a 100 mL round-bottom flask, and tetrabutylammonium fluoride (49 mL) was added. The reaction mixture was stirred at room temperature overnight. Upon completion, the solvent was evaporated, and the crude product was purified by column chromatography (hexane/ethyl acetate = 2:1, v/v) to afford Compound 7 as a colorless oil (1.092 g, 59.2%).

**Synthesis of DRCy.** Compound 7 (150 mg, 0.39 mmol) was dissolved in DCM. Et₃N (82.9 mg, 0.82 mmol) and a catalytic amount of DMAP were added. The mixture was cooled to 0 °C, and a solution of PNP chloroformate (165.3 mg, 0.82 mmol) in 20 mL DCM was added dropwise. The reaction was stirred at room temperature for 16 h. The solvent was removed under reduced pressure, and the residue was redissolved in DCM. A solution of CyOH (326.7 mg, 0.82 mmol) in 10 mL DCM was then added dropwise at 0 °C. After the addition, the mixture was warmed to room temperature and stirred overnight. Upon completion, the solvent was evaporated, and the residue was purified by silica gel column chromatography using dichloromethane/methanol (100:1 to 10:1, v/v) as the eluent to afford the desired product as a blue powder (80 mg, yield 53%).

**Scheme S4.** Synthetic procedures of QRCy.

**Synthesis of Compound 8.** Compound 5 (2 g, 1.2 mmol) was dissolved in 15 mL DCM, and Et₃N (1.324 g, 1.3 mmol) along with a catalytic amount of DMAP were added. The reaction mixture was cooled to 0 °C, and a solution of PNP chloroformate (2.636 g, 1.3 mmol) in 10 mL DCM was added dropwise. The reaction was stirred at room temperature for 16 h. The solvent was removed under reduced pressure, and the residue was redissolved in DMF, followed by the addition of N-Boc-N,N'-dimethylethylamine (3.765 g, 5.9 mmol). The reaction was stirred at room temperature for 50 minutes and monitored by TLC (hexane/ethyl acetate = 5:1, v/v). Upon completion, the solvent was removed under reduced pressure, and the crude product was purified by silica gel column chromatography (hexane/ethyl acetate = 5:1, v/v) to afford Compound 8 as an oil (380 mg, yield 60%).

**Synthesis of Compound 9.** Compound 7 (300.0 mg, 0.8 mmol) was dissolved in 10 mL DCM, and Et_3_N (166.0 mg, 1.6 mmol) along with a catalytic amount of DMAP were added. The reaction mixture was cooled to 0°C, and a solution of PNP chloroformate (330.1 mg, 1.6 mmol) in 5 mL DCM was added dropwise. The reaction was stirred at room temperature for 16 h. The solvent was removed under reduced pressure, and the residue was redissolved in DMF. Compound 8 (860.5 mg, 1.4 mmol) was deprotected with 2 ml TFA to remove the Boc group. The excess of the acid was removed under reduced pressure and the residue was dissolved in 2 mL DMF, followed by the addition of the above solution. The reaction was stirred at room temperature for 1 h and monitored by TLC (hexane/ethyl acetate = 2:1, v/v). Upon completion, the solvent was removed under reduced pressure, and the crude product was purified by silica gel column chromatography (hexane/ethyl acetate = 2:1, v/v) to afford Compound 9 as a yellow oil (420 mg, yield 36%).

**Synthesis of Compound 10.** Compound 9 (1 g, 0.7 mmol) was dissolved in tetrahydrofuran (10 mL) in a 100 mL round-bottom flask, and tetrabutylammonium fluoride (6.859 mL) was added. The reaction mixture was stirred at room temperature overnight. Upon completion, the organic solvent was removed, and the crude product was purified by column chromatography (dichloromethane/methanol = 10:1, v/v) to afford Compound 10 as a yellow oil (308.5 mg, yield 44%).

**Synthesis of Compound QRCy.** Compound 10 (150 mg, 0.15 mmol) was dissolved in DCM. Et_3_N (62.2 mg, 0.61 mmol) and a catalytic amount of DMAP were added. The reaction mixture was cooled to 0 °C, and a solution of PNP chloroformate (123.8 mg, 0.61 mmol) in 6 mL DCM was added dropwise. The reaction was stirred at room temperature for 18 h. The solvent was removed under reduced pressure, and the residue was redissolved in DCM. A solution of CyOH (244.9 mg, 0.61 mmol) in 15 mL DCM was then added dropwise at 0 °C. After the addition, the reaction mixture warmed to room temperature and stirred overnight. Upon completion, the solvent was evaporated, and the residue was purified by silica gel column chromatography using dichloromethane/methanol (100:1 to 10:1, v/v) as the eluent to afford the desired product as a blue powder (108 mg, yield 26.6%).

**Synthesis of PAMAM-Ser conjugates.** Boc-Ser-OH (34.5 mg, 0.132 mmol) was dissolved in 5 mL of DMSO, and 1.2 equivalents of HBTU (39.2 mg, 0.106 mmol), HOBt (14.0 mg, 0.106 mmol), and DIPEA (30.7 μL, 0.176 mmol) were added simultaneously. After stirring for 4 h, PAMAM (G5) dendrimer (63.4 mg, 0.0022 mmol) was added to the above solution, and the reaction was allowed to proceed overnight. The product was then purified by three rounds of ether precipitation. The resulting product was dissolved in a TFA mixture (95% TFA, 2.5% TIS, and 2.5% water) and stirred at room temperature for 90 minutes to deprotect the Boc group. The product was then purified again by three rounds of ether precipitation. By varying the feed ratio of PAMAM to Boc-Ser-OH (1:60, 1:100, and 1:140), three different PAMAM-Ser conjugates with high, medium, and low serine grafting ratios were synthesized, denoted as PS1, PS2, and PS3, respectively. The grafting rate of L-serine in PS1, PS2, and PS3 was calculated by integrating the integral ratio of the methylene protons of Ser to the methylene protons (CONHCH_2_) of PAMAM in the ¹H NMR spectrum [1].

***In vivo* pharmacokinetics and tissue distribution of PS-QRC.** To track the in vivo metabolism, the Fluorescein isothiocyanate (FITC)-labeled nanoprobe FITC-PS-QRC was intravenously injected into the mice. Blood samples were collected at predetermined time points ranging from 0 to 12 h post-injection. The probe concentration in the plasma was quantified and converted to % of dose/mL. A two-phase decay nonlinear regression model was employed to fit the complete 0-12 h concentration-time curve to calculate the terminal elimination half-life (t_1/2_). Simultaneously, the area under the local concentration-time curve from 0 to 2 h (AUC_0-2h_) was calculated. Major organs were harvested at 2 h post-injection to extract and quantify the absolute tissue accumulation (% ID).

**Intra-renal microdistribution imaging of the nanoprobe.** To observe the Probe distribution of PS-QRC within the renal microstructure, the PS-QRC was intravenously injected into the mice. Two hours post-injection, the intact kidneys were excised, fixed in paraformaldehyde, dehydrated in sucrose, and then sectioned into 5-μm-thick cryosections. Subsequently, the sections were stained with DAPI for nuclear visualization. The precise microdistribution of CyOH within the renal cortex and medulla was acquired and analyzed using an automated quantitative pathology imaging system (TissueFAXS).

References

[1]	a) W. G. Lesniak, N. Oskolkov, X. Song, B. Lal, X. Yang, M. Pomper, J. Laterra, S. Nimmagadda, M. T. McMahon, *Nano Lett* **2016**, 16, 2248; b) S. Matsuura, H. Katsumi, H. Suzuki, N. Hirai, H. Hayashi, K. Koshino, T. Higuchi, Y. Yagi, H. Kimura, T. Sakane, A. Yamamoto, *Proc Natl Acad Sci U S A* **2018**, 115, 10511.

**Figure S1**. ^1^H NMR spectra of compound 1 in DMSO-*d6*.

**Figure S2**. ^1^H NMR spectra of compound 2 in DMSO-d6.

**Figure S3**. ^1^H NMR spectra of compound 2 in DMSO-d6.

**Figure S4**. ^1^H NMR spectra of CyOH in CDCl_3_.

**Figure S5**. ^13^C NMR spectra of CyOH in DMSO-*d6*.

**Figure S6**. Mass spectrometry (MS) analysis of CyOH.

**Figure S7**. ^1^H NMR spectra of compound 4 in CDCl_3_.

**Figure S8**. ^1^H NMR spectra of RCy in CDCl_3_.

**Figure S9**. ^13^C NMR spectra of RCy in DMSO-*d6*.

**Figure S10**. MS analysis of RCy.

**Figure S11**. ^1^H NMR spectra of compound 5 in CDCl_3_.

**Figure S12**. ^1^H NMR spectra of compound 6 in CDCl_3_.

**Figure S13**. ^1^H NMR spectra of compound 7 in CDCl_3_.

**Figure S14**. ^1^H NMR spectra of DRCy in DMSO-*d6*.

**Figure S15**. ^13^C NMR spectra of DRCy in DMSO-*d6*.

**Figure S16**. MS analysis of DRCy.

**Figure S17**. ^1^H NMR spectra of compound 8 in CDCl_3_.

**Figure S18**. ^1^H NMR spectra of compound 10 in CDCl_3_.

**Figure S19**. ^1^H NMR spectra of QRCy in methanol-*d4*.

**Figure S20**. ^13^C NMR spectra of QRCy in CDCl_3_.

**Figure S21**. MS analysis of QRCy.

**Figure S22**. UV-vis absorption (A) and fluorescence emission spectroscopy (B) of CyOH.

**Figure S23**. Fluorescence responses of RCy (A) and DRCy (B) toward various analytes. (C) Fluorescence responses of QRCy toward various ROS. Data are presented as the mean ± SD, *n* = 3. Statistical significance was calculated *via* one-way ANOVA. ****p* < 0.001.

**Figure S24**. (A) Representative fluorescence microscopy images of HK-2 cells that were previously stimulated with cisplatin and then incubated with PS-Cy, the cells were pretreated with or without KIM-1 (30 μg/ml) antibody for 1 hour before administration of PS-Cy. (B) Quantitative analysis of fluorescence intensity of CyOH in HK-2 cells after different treatments. Data are presented as the mean ± SD, *n* = 3. Statistical significance was calculated *via* one-way ANOVA. ****p* < 0.001.

**Figure S25**. ^1^H NMR spectra of PAMAM-Ser conjugates in D_2_O.

**Figure S26**. The size (A) and PDI (B) changes of PS-QRC after incubation in PBS or PBS containing 10% fetal bovine serum (FBS) for 36 h. (C) The fluorescence intensity changes of PS-QRC after incubation in PBS or PBS containing 10% FBS for 36 h. Data are presented as mean values ± SD, *n* = 3.

**Figure S27**. Cell viability of HK-2 cells (A), 3T3 cells (B), and HUVECs (C) treated with different concentrations of PS-RC, PS-DRC, and PS-QRC for 24 h (*n* = 5). (D) Relative intracellular ROS levels in HK-2 cells after treatment with different concentrations of PS-RC, PS-DRC, and PS-QRC for 24 h (*n* = 3). Data are presented as the mean ± SD, *n* = 3-5.

**Figure S28**. (A) Time courses of plasma concentration of PS-QRC in mice after *i.v.* injection at a dose of 1 mg/kg. (B) High performance liquid chromatography (HPLC) quantitative analysis of residual RP-SC in major organs 2 hours after intravenous injection. Data are presented as the mean ± SD, *n* = 3.

**Figure S29**. The distribution of activated PS-QRC in renal tissue sections at 2 hours after i.v. injection in mice.

**Figure S30.** Calculated SBR values corresponding to Figure 4F. Data are presented as the mean ± SD; n = 3. Statistical significance was calculated *via* one-way ANOVA. ****p* < 0.001.

**Figure S31.** (A) Representative *ex vivo* fluorescence images of kidneys harvested from AKI model mice at 2 h post-intravenous injection of PS-RC, PS-DRC, and PS-QRC. (B) Quantitative analysis of fluorescence intensities in kidneys (*n* = 3). Data are presented as the mean ± SD; *n* = 3. An unpaired two-tailed t-test was used in panels (B). *** *p* < 0.001.

**Figure S32.** Levels of kidney-liver function markers in healthy mice administrated with PS-QRC after 24 h. Kidney-liver function markers contain ALT (A), ALB (B), BUN (C), UA (D). Data are presented as the mean ± SD; *n* = 3. An unpaired two-tailed t-test was used in panels (A-D). ns p > 0.05.

**Figure S33.** H&E staining images of the main organs from mice after intravenous injected with PS-QRC.

**Figure S34.** Survival of mice receiving various treatments.
